# Effectiveness of Indirect and Direct Laryngoscopes in Pediatric Patients: A Systematic Review and Network Meta-Analysis

**DOI:** 10.3390/children9091280

**Published:** 2022-08-25

**Authors:** Hiroshi Hoshijima, Takahiro Mihara, Shinichi Kokubu, Sakura Takeda, Toshiya Shiga, Kentaro Mizuta

**Affiliations:** 1 Division of Dento-Oral Anesthesiology, Graduate School of Dentistry, Tohoku University, Sendai 980-8575, Japan; 2 Department of Health Data Science, Graduate School of Data Science, Yokohama City University, Yokohama 236-0004, Japan; 3 Department of Anesthesiology, Dokkyo Medical University, 880 Kitakobayashi, Mibu 321-0293, Japan; 4 Department of Anesthesiology and Pain Medicine, International University of Health & Welfare Ichikawa Hospital, 6-1-4 Kounodai, Ichikawa 272-0827, Japan

**Keywords:** indirect laryngoscopes, direct laryngoscopes, pediatric, network meta-analysis

## Abstract

This research aimed to produce a coherent ranking of the effectiveness of intubation devices in pediatric patients using network meta-analysis (NMA). We searched the electric databases for prospective randomized studies that compared different tracheal intubation devices in pediatric patients. The primary outcome was intubation failure at the first attempt. Secondary outcomes were glottic visualization and intubation time. The statistical analysis performed used DerSimonian and Laird random-effects models. Frequentist network meta-analysis was conducted, and network plots and network league tables were produced. Subgroup analysis was performed after excluding rigid-fiberscope-type indirect laryngoscopes. Thirty-four trials comparing 13 devices were included. Most laryngoscopes had the same intubation failure rate as the Macintosh reference device. Only the Truview PCD™ had a significantly higher intubation failure rate than the Macintosh (odds ratio 4.78, 95% confidence interval 1.11–20.6) The highest-ranking laryngoscope was the Airtaq™ (P score, 0.90), and the AirwayScope™, McGrath™, and Truview EVO2™ ranked higher than the Macintosh. The Bullard™ had the lowest ranking (P score, 0.08). All laryngoscopes had the same level of glottic visualization as the Macintosh and only the C-MAC™ had a significantly shorter intubation time. Intubation time was significantly longer when using the GlideScope™, Storz DCI™, Truview PCD™, or Bullard™ compared with the Macintosh. P score and ranking of devices in the subgroup analyses were similar to those in the main analysis. We applied NMA to create a consistent ranking of the effectiveness of intubation devices in pediatric patients. The findings of NMA suggest that there is presently no laryngoscope superior to the Macintosh laryngoscope in terms of tracheal intubation failure rate and glottic visualization in pediatric patients.

## 1. Introduction

Tracheal intubation in pediatric patients is difficult because the anatomy of the oral cavity, glottis, and trachea is smaller than that in adults. Furthermore, the laryngoscopes and tracheal tubes used for tracheal intubation in children are smaller than those in adults, and their use requires special skill [1,2]. Therefore, there is an increased risk of complications, such as hypoxia, bradycardia, and tachycardia, when performing tracheal intubation in pediatric patients [3,4,5].

A conventional direct laryngoscope, such as the Macintosh laryngoscope or Miller laryngoscope, is usually used for tracheal intubation in pediatric patients. Various types of indirect laryngoscopes are also now clinically used for tracheal intubation in these patients. Tracheal intubation with a conventional laryngoscope requires the laryngoscopist to bring the oral, pharyngeal, and laryngeal axes into a straight line extending from the incisors to the larynx. An indirect laryngoscope incorporates a digital camera in the tip of the blade that displays an image of the glottis on an external monitor. This structural feature of indirect laryngoscopes may aid tracheal intubation in pediatric patients. Indirect laryngoscopes are considered advantageous for tracheal intubation because they allow a clearer view of the small glottis and epiglottis in the narrow oral cavity of a pediatric patient on an external monitor [6].

Various studies of intubation using indirect laryngoscopes in pediatric patients have been reported. Previous pairwise meta-analyses have found that intubation times are longer in pediatric patients when an indirect laryngoscope is used, although the success rate is not significantly different from that achieved with a direct laryngoscope [7,8]. However, it is not known which of the indirect laryngoscopes is the best tracheal device for pediatric patients.

The aim of this research was to produce a coherent ranking of the effectiveness of intubation devices in pediatric patients using network meta-analysis (NMA).

## 2. Methods

### 2.1. Protocol and Registration

This review was prepared according to the recommendations of the Preferred Reporting Items for Systematic Reviews and Meta-Analyses (PRISMA) extension statement for reporting systematic reviews incorporating network meta-analysis (PRISMA-NMA) [9]. The study protocol was registered in PROSPERO (registration number: CRD42021260230).

### 2.2. Search Strategy

We performed a comprehensive search of the PubMed, EMBASE, and Cochrane Central Register of Controlled Trials databases. The search strategy is shown in Figure S1. We also manually searched the reference lists in the reports and reviews extracted. No restrictions were placed on the language of publication or type of article. The search was performed in August 2021.

### 2.3. Study Selection and Data Collection

We searched for articles using the formula shown in Figure S1. Potentially eligible articles were extracted and assessed by two of the authors (H.H., T.S.) working independently of each other. Disagreements regarding interpretation or analysis of data in the extracted articles were resolved by discussion. If there were reports of the same or updated data, only the report analyzing the latest data was added to the meta-analysis. Authors were contacted directly in the event of missing data or data inconsistencies. We also searched registry websites to determine if the research protocol for each study included in this meta-analysis had been published in advance, and, if so, we confirmed that it matched the research content; if not, a risk of bias was recorded.

### 2.4. Eligibility Criteria

Studies were eligible for inclusion if they had a prospective randomized design and compared tracheal intubation devices in pediatric patients. Trials involving flexible fiber intubation, MaCoy laryngoscope, manikins, and tracheal intubation performed during cardiopulmonary resuscitation were excluded. Furthermore, we excluded studies in which the type of laryngoscope used was unclear or in which two different laryngoscopes were used in the same patient. The following data were extracted: intubation failure at the first attempt, glottic visualization (Cormack–Lehane classification 1 vs. 2 or higher), and intubation time. We used the definitions in each study for tracheal intubation failure and intubation time.

The primary outcome was intubation failure at the first attempt. The secondary outcomes were glottic visualization and intubation time.

### 2.5. Risk of Bias within Individual Studies

We evaluated the risk of bias for each individual study with reference to the Cochrane Handbook for Systematic Reviews of Interventions [10] (Figure S2). Risk of bias was evaluated independently by the two reviewers (H.H., T.S.).

### 2.6. Certainty of Evidence

The Grading of Recommendations, Assessment, Development, and Evaluation (GRADE) approach was used to rate the quality of evidence for each network estimate [11]. In this approach, the rating of direct evidence from randomized controlled trials (RCTs) starts at “high” quality and can be described as “moderate”, “low”, or “very low” based on the following six domains: within-study bias, across-studies bias, indirectness, imprecision, heterogeneity, and incoherence (inconsistency). A node-splitting model was used to evaluate inconsistency between direct and indirect estimates [12]. We used the Confidence In Network Meta-Analysis (CINeMA) web application based on the previously developed framework [13].

### 2.7. Publication Bias

We produced a comparison-adjusted funnel plot to detect possible small-study effects on the primary outcome. Before using this plot, we anticipated that new treatments would be preferred and, therefore, we arranged the laryngoscopes from old to new so that all comparisons would refer to “old” and “new” interventions. We then calculated the difference in study-specific effect sizes.

### 2.8. Sensitivity Analysis

For the sensitivity analysis, risk was assessed using the Risk-of-Bias tool. Only trials with a low risk of bias were selected for meta-analysis. The outcomes analyzed were intubation failure, glottic visualization, and intubation time.

### 2.9. Subgroup Analysis

Subgroup analysis was performed after excluding rigid-fiberscope-type indirect laryngoscopes (Bonfils and Bullard) because of the difference in operability between those laryngoscopes and blade-type indirect laryngoscopes.

### 2.10. Statistical Analysis

The statistical analysis was performed using DerSimonian and Laird random-effects models. The pooled effect estimates of binary variables (intubation failure, glottic visualization) are expressed as the odds ratio (OR) with the 95% confidence interval (CI). The pooled difference in intubation time between each type of intubation device is expressed as the mean difference (MD) of the 95% CI.

The P score was used to rank the effectiveness of each device and identify the best device in terms of each outcome [14]. The P score ranges from 0 to 1, and the closer it is to 1, the higher the probability that an intervention ranks first or is in one of the top ranks. A frequentist NMA was performed using R version 4.0.5 (R Foundation for Statistical Computing, Vienna, Austria) with the netmeta package version 8 × 10^4^ package. A random-effects model was used when pooling effect size.

## 3. Results

### 3.1. Characteristics of the Studies in the Meta-Analysis

The initial search of the above-mentioned electronic databases yielded 401 potentially relevant articles, and 165 unrelated studies were excluded based on their titles and abstracts. We then carefully read the full text of the remaining 236 articles to determine whether they met the inclusion and exclusion criteria. A further 202 studies were excluded for the following reasons: non-RCT (n = 56), manikin study (n = 41), case report (n = 33), review or meta-analysis (n = 22), different outcomes (n = 16), use of a flexible fiberscope (n = 11), use of a laryngeal mask (n = 7), observational study design (n = 7), adult patients (n = 4), guidelines (n = 3), and resuscitation research (n = 3). The remaining 34 articles met the inclusion criteria and contained the data necessary for the planned analysis (Figure S3) [15,16,17,18,19,20,21,22,23,24,25,26,27,28,29,30,31,32,33,34,35,36,37,38,39,40,41,42,43,44,45,46,47].

All studies included in the meta-analysis were published between 2008 and 2020. Patients included in these studies were aged 0 to 10 years. Based on pre-intubation airway assessment, 26 of 34 studies included patients with a normal airway [15,16,17,20,22,23,24,25,26,27,28,29,30,31,32,33,37,38,39,40,41,42,43,46,47], and 3 included patients with a difficult airway [35,36,45]. Five studies did not provide information on airway status [18,19,21,34,44]. The 34 trials investigated 13 types of laryngoscope, namely, the Macintosh (used in 25 trials), GlideScope™ (n = 10, GlideScope), Miller (n = 8), Airtraq™ (n = 5, Airtraq), Airway Scope™ (n = 5, AWS), McGrath™ (n = 5, McGrath), C-MAC™ (n = 4, C-MAC), Truview EVO2™ (n = 3, Truview EVO2), Truview PCD™ (n = 2, Truview PCD), King Vision™ (n = 2, King Vision), Storz DCI™ (n = 2, Storz DCI), Bullard™ (n = 1, Bullard), and Bonfils™ (n = 1, Bonfils) (Table 1).

### 3.2. Risk of Bias

**Figure 1:**
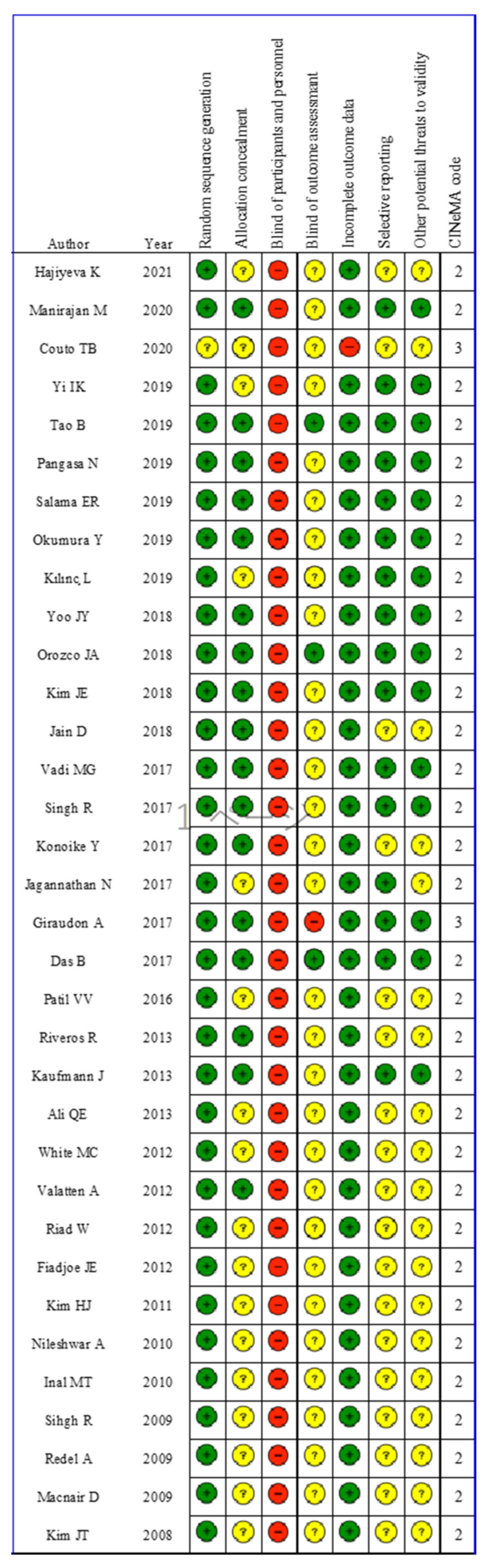
The risk of bias assessment. Green circles, red circles, and yellow circles indicate “low risk of bias”, “high risk of bias”, and “unclear risk of bias”, respectively.

Figure 1 summarizes the risk of bias. Most of the studies included random sequence generation and explained any withdrawals and missing data. However, blinding of the laryngoscope used was not possible in any of the studies. Moreover, in most studies, the assessing physician was not blinded. The majority of the more recent studies had been pre-registered online, but some of the earlier studies were not pre-registered.

### 3.3. Primary Outcome

#### Intubation Failure at First Attempt 

**Figure 2:**
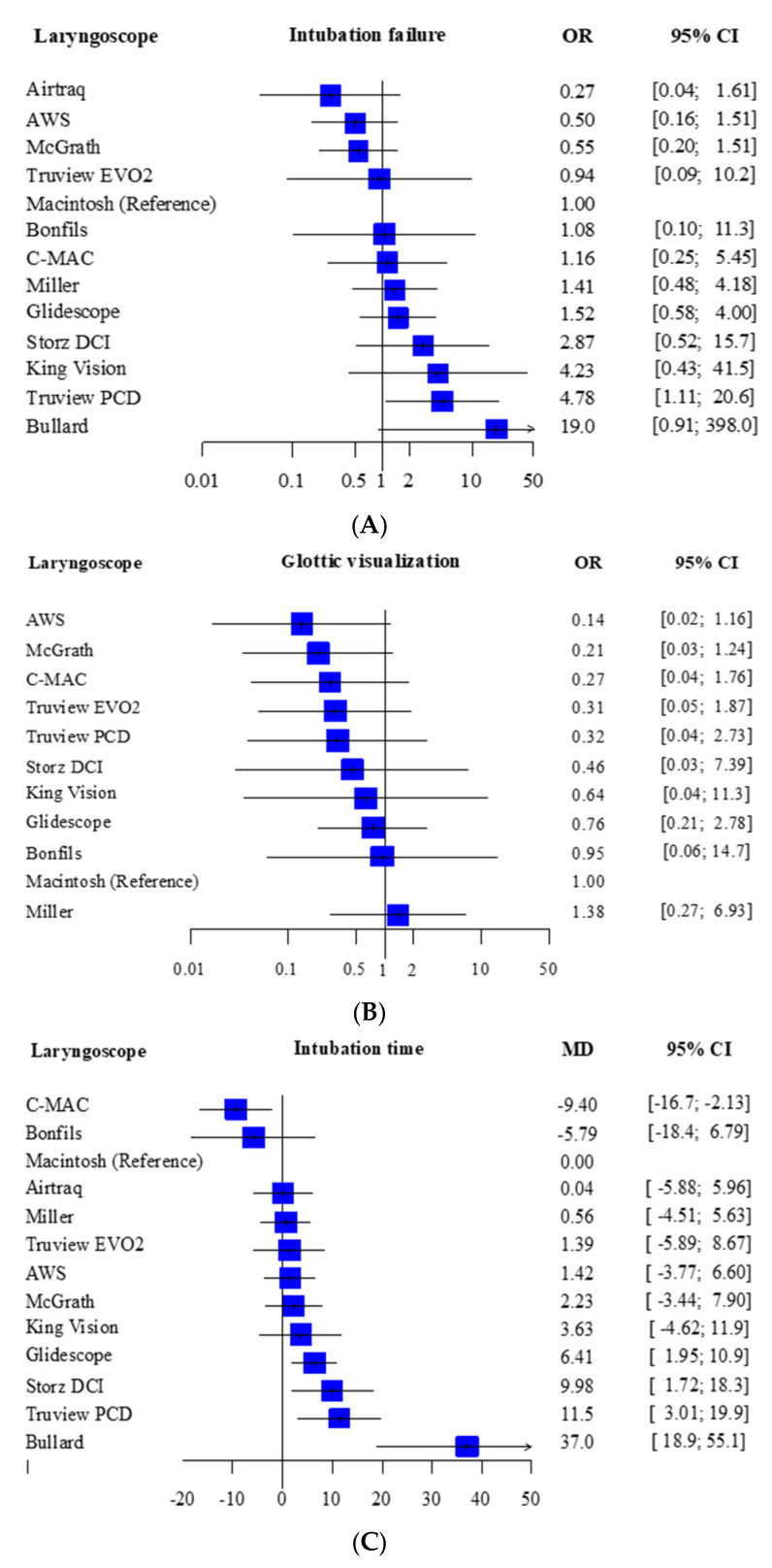
Forest plot of the main results. (**A**) Forest plot of the intubation failure of tracheal intubation using the indirect laryngoscope compared with the Macintosh laryngoscope. (**B**) Forest plot of glottic visualization with the indirect laryngoscope compared with the Macintosh laryngoscope, Cormack–Lehane grade 1 and 2 vs. other grades. (**C**) Forest plot of intubation time for tracheal intubation using the indirect laryngoscope compared with the Macintosh laryngoscope.

**Figure 3:**
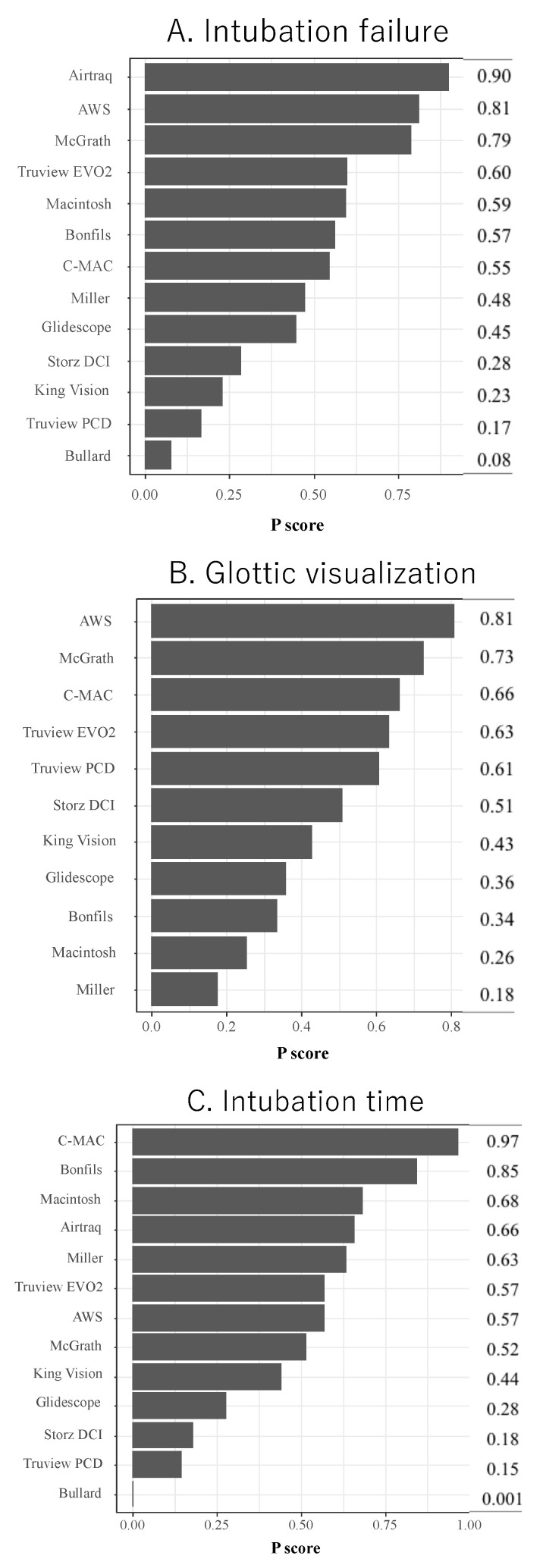
The results of primary (**A**) and secondary outcome (**B**,**C**) by P score ranking.

Intubation failure could be investigated in 14 studies that included 1322 patients. Twenty-one studies and 1930 patients were compared by direct comparison and indirect comparison of NMA. Most laryngoscopes had the same intubation failure rate as the Macintosh; only the Truview PCD had a significantly higher intubation failure rate (OR 4.78, 95% CI 1.11–20.6) (Figure 2A). The expected P scores and ranking of each intubation device in terms of intubation failure at the first attempt are shown in Figure 3A. The highest-ranked laryngoscope was the Airtaq (P score, 0.90), and the AWS, McGrath, and Truview EVO2 ranked higher than the Macintosh. The Bullard had the lowest ranking (P score, 0.08). A league table summarizing the results for intubation failure is presented in Figure S4. A summary of the findings for the primary outcome is provided in Table 2.

CINeMA did not find any evidence of inconsistency between direct and indirect comparisons (Figure S5). The comparison-adjusted funnel plot and Egger’s test results indicate that there was no publication bias (*p* = 0.31) (Figure S6A).

### 3.4. Secondary Outcome

#### Glottic Visualization and Intubation Time

Glottic visualization was analyzed in 10 studies (813 patients). Sixteen studies and 1459 patients were compared by direct comparison and indirect comparison of NMA. All laryngoscopes had the same level of glottic visualization as the Macintosh. (Figure 2B) Figure 3B shows the expected P scores and ranks the ability of each intubation device to visualize the glottis. Glottic visualization was highest for the Airway Scope (P score, 0.81) and lowest for the Miller device (P score, 0.17). All indirect laryngoscopes ranked higher than the Macintosh (Figure S7). CINeMA found no evidence of inconsistency between direct and indirect comparisons (Figure S8). The comparison-adjusted funnel plot and Egger’s test results indicate there was no publication bias (*p* = 0.67) (Figure S6B). The findings for glottic visualization are shown in Figure S9.

Intubation time was analyzed in 25 studies (2142 patients). Thirty-one studies and 2599 patients were compared by direct comparison and indirect comparison of NMA. Analysis with reference to the Macintosh showed that only the C-MAC had a significantly shorter intubation time (–MD –9.40, 95% CI–16.7, –2.13). The intubation time was significantly longer for the GlideScope (MD 6.41, 95% CI 1.95–10.9), Storz DCI (MD 9.98, 95% CI 1.72–18.3), Truview PCD (MD 11.5, 95% CI 3.01–19.9), and Bullard (MD 37.0, 95% CI 18.9–55.1) compared with the Macintosh (Figure 2C). The expected P score and ranking of each device in terms of intubation time are shown in Figure 3C. Of all the laryngoscopes, the one with the shortest intubation time was the C-MAC (P score, 0.97) (Figure S10). CINeMA found no evidence of inconsistency between direct and indirect comparisons (Figure S11). The comparison-adjusted funnel plot and Egger’s test results indicate that there was no publication bias (*p* = 0.11) (Figure S6C). The intubation time data are shown in Figure S12. The Airtraq, AWS, and McGrath devices were ranked highest for intubation failure and glottic visualization. However, the intubation time for these laryngoscopes was no shorter than that for the Macintosh.

### 3.5. Sensitivity Analysis

The laryngoscopes used in the included studies could not be blinded. Therefore, no study was low-risk according to the GRADE evaluation, and sensitivity analysis could not be performed.

### 3.6. Subgroup Analyses

Except for the Bonfils and Bullard devices, the P score and ranking of devices in the subgroup analyses were similar to those in the main analysis (Figures S13 and S14A–C). The comparison-adjusted funnel plot and Egger’s test results indicate that there was no publication bias in intubation failure, glottis visualization, and intubation time (Figure S15A–C).

## 4. Discussion

The findings of this systematic review and NMA suggest that none of the currently available laryngoscopes are significantly better than the Macintosh device in terms of intubation failure and glottic visualization. On the other hand, the intubation time was significantly shorter for only the C-MAC indirect laryngoscope and significantly longer for the GlideScope, Storz DCI, Truview PCD, and Bullard devices compared with the Macintosh laryngoscope.

In terms of P scores, the AWS, McGrath, and Truview EVO2 laryngoscopes ranked higher for both intubation failure and glottic visualization. Glottic visualization with the Airtraq could not be assessed because there were no relevant RCTs available for inclusion in the NMA. The P score rankings for intubation failure and glottic visualization were similar in the subgroup analyses. However, among the laryngoscopes with the highest intubation failure ranking, only the Airtraq ranked higher for intubation time. These findings suggest that the Airtraq is the most useful device for tracheal intubation in pediatric patients. The AWS, McGrath, and Truview EVO2 were ranked higher for intubation failure and glottic visualization; these laryngoscopes were ranked equal to or lower than the Macintosh in intubation time.

The present study compared and analyzed the glottic visualization of indirect laryngoscopes and direct laryngoscopes. Glottic visualization was better with all indirect laryngoscopes than with direct laryngoscopes in regard to P scores. However, not all indirect laryngoscopes improved intubation failure significantly more than direct laryngoscopes. Conventionally, the grade of glottic visualization has been regarded as an important factor for the success of tracheal intubation [48]. However, when indirect laryngoscopy began to be used, it was proven that the success rate of tracheal intubation did not necessarily increase even with excellent glottal visualization [49,50]. The reason for this is that, even when using an indirect laryngoscope, it is necessary to operate the tracheal tube while looking at the monitor screen. This maneuver requires eye–hand coordination and can be challenging compared to using a direct laryngoscope. Second, indirect laryngoscopes can see the glottis with less laryngeal force than direct laryngoscopes. Therefore, it is necessary to operate the intubation tube in a narrow oral cavity. However, it is difficult to guide the tracheal tube to the glottis because the working space of the intubation tube is narrow in the narrow oral cavity.

The Bonfils and Bullard laryngoscopes are believed to have longer intubation times. In our NMA, compared with the Macintosh device, the intubation time was significantly longer with the Bullard, which was ranked last by P score, but not with the Bonfils, which was ranked second. This result indicates that the shape of the intubation device alone cannot determine its usefulness in pediatric patients. However, our NMA contained only one RCT that compared the Bonfils and Bullard devices, so further research is needed.

In this NMA, the indirect laryngoscope most often studied in the RCTs was the Glidescope. There was no significant difference in the intubation failure rate or glottic visualization between the Glidescope and Macintosh. However, the intubation time was significantly longer with the Glidescope. Moreover, the Glidescope had a low P score ranking in all analyses of intubation failure, glottic visualization, and intubation time. Of note, much of the research on the Glidescope was carried out in the early years of research on indirect laryngoscopes (2008–2013), and the low evaluation score for this device, particularly for the intubation time, may reflect anesthesiologists’ lack of familiarity with the use of indirect laryngoscopes [51,52]. Furthermore, when intubating the trachea using the Glidescope, it is necessary to bend the stylet by at least 50–60 degrees so that it matches the angle of the blade, which would also increase the intubation time [18].

This NMA has several limitations. First, the laryngoscope used for research cannot be blinded, which increases the likelihood of bias. Second, the patients in each RCT varied in age from 0 to 10 years. Children gain height and weight as they grow, so the difficulty of tracheal intubation varies according to age, as does the size of the laryngoscope blade used. These variations have implications in terms of the quality of the study. Third, pre-intubation airway status varied from study to study. Our meta-analysis included 25 trials in 33 patients with a normal airway, 3 patients with a difficult airway, and 5 whose airway status was unknown. A subgroup analysis of patients in whom intubation was difficult had been planned but could not be performed because the number of trials was too small. Finally, there were wide variations in the characteristics of the study populations and the dosages of anesthetic agents used, which are further sources of bias.

We applied a network meta-analysis (NMA) to create a consistent ranking of the effectiveness of intubation devices in pediatric patients. In conclusion, the findings of this systematic review and NMA suggest that there is presently no laryngoscope superior to the Macintosh laryngoscope in terms of tracheal intubation failure rate and glottic visualization in pediatric patients. However, the intubation time was significantly shorter when using the C-MAC indirect laryngoscope than when using the Macintosh. The intubation time was significantly longer for the GlideScope, Storz DCI, Truview PCD, and Bullard devices than for the Macintosh. The Airtraq, AWS, and McGrath ranked higher for intubation failure and glottic visualization, but these laryngoscopes were ranked equal to or lower than the Macintosh in intubation time. Except for the Bonfils and Bullard devices, the P score and ranking of devices in the subgroup analyses were similar to those in the main analysis.

## Figures and Tables

**Table 1:** Patients Characteristics.

	Author	Year	Type of Laryngoscopes	Number of Participants	Patients Age or Weight	ASA-PS	Airway Condition
1	Hajiyeva K	2021	Macintosh	28	10–40 kg	Ⅰ–Ⅲ	Normal
			C-MAC	28			
2	Manirajan M	2020	Macintosh	39	0–1 y	Ⅰ–Ⅱ	Normal
			King Vision	39			
3	Couto TB	2020	Macintosh	141	1–19 y	N/A	Difficult (Emergency department)
			McGrath	50			
4	Yi IK	2019	Macintosh	68	1–10 y	Ⅰ–Ⅱ	Normal
			AWS	68			
5	Tao B	2019	Macintosh	35	≦28 d	Ⅰ–Ⅱ	No limitation
			Glidescope	35			
6	Pangasa N	2019	Macintosh	25	2–8 y	Ⅰ–Ⅱ	Normal
			Truview EVO2	25			
7	Salama ER	2019	Miller	30	≦28 d	Ⅰ–Ⅱ	Normal
			Glidescope	30			
8	Okumura Y	2019	Macintosh	20	3–11 m	Ⅰ	Normal
			AWS	20			
9	Kılınç L	2019	Macintosh	40	1–12 y	Ⅰ–Ⅱ	Normal
			Glidescope	40			
10	Yoo JY	2018	Macintosh	36	1–10 y	Ⅰ–Ⅱ	Normal
			AWS	35			
			McGrath	35			
11	Orozco JA	2018	Macintosh	40	2–8 y	Ⅰ–Ⅱ	Normal
			AWS	40			
12	Kim JE	2018	Macintosh	42	1–10 y	Ⅰ–Ⅱ	Normal
			McGrath	42			
13	Jain D	2018	Miller	32	<1 y	Ⅰ–Ⅲ	Difficult (lateral position)
			C-MAC	31			
14	Vadi MG	2017	Miller	31	2–24 m	Ⅰ–Ⅲ	Difficult (MILS)
			Glidescope	31			
			Storz DCI	31			
15	Singh R	2017	Macintosh	50	1–6 y	Ⅰ–Ⅱ	No limitation
			C-MAC	50			
			Truview PCD	50			
16	Konoike Y	2017	Miller	29	1.7–6 y	Ⅰ–Ⅱ	Normal
			AWS	30			
			McGrath	31			
17	Jagannathan N	2017	Miller	100	<2 y	N/A	Normal
			King Vision	100			
18	Giraudon A	2017	Macintosh	67	10–20 kg	Ⅰ–Ⅱ	Normal
			McGrath	65			
19	Das B	2017	Miller	30	2–10 y	Ⅰ–Ⅱ	Normal
			Airtraq	30			
20	Patil VV	2016	Macintosh	30	8–18 y	Ⅰ–Ⅱ	Normal
			C-MAC	30			
21	Riveros R	2013	Macintosh	45	0–10 y	Ⅰ–Ⅲ	Normal
			Glidescope	44			
			Truview PCD	45			
22	Kaufmann J	2013	Glidescope	47	<7 y	Ⅱ	Normal
			Bonfils	44			
23	Ali QE	2013	Macintosh	17	1–5 y	Ⅰ–Ⅱ	Normal
			Airtraq	17			
24	White MC	2012	Macintosh	30	0–6 m, 6 m–6 y	Ⅰ–Ⅱ	Normal
			Airtraq	30			
25	Valatten A	2012	Macintosh	25	≦5 y	N/A	Normal
			Airtraq	24			
26	Riad W	2012	Macintosh	25	2–10 y	Ⅰ	Normal
			Airtraq	25			
27	Fiadjoe JE	2012	Macintosh	30	≦12 y	Ⅰ–Ⅱ	Normal
			Glidescope	30			
28	Kim HJ	2011	Macintosh	40	<10 y	Ⅰ–Ⅱ	Normal
			Glidescope	40			
29	Nileshwar A	2010	Macintosh	20	2–10 y	Ⅰ–Ⅱ	Normal
			Bullard	20			
30	Inal MT	2010	Miller	25	2–8 y	N/A	N/A
			Truview EVO2	25			
31	Sihgh R	2009	Miller	30	1–10 kg	N/A	N/A
			Truview EVO2	30			
32	Redel A	2009	Macintosh	30	7 m–10 y	Ⅰ–Ⅲ	Normal
			Glidescope	30			
33	Macnair D	2009	Macintosh	30	2–16 y	Ⅰ–Ⅱ	Normal
			Storz DCI	30			
34	Kim JT	2008	Macintosh	100	3 m–17 y	N/A	No limitation
			Glidescope	103			

y: year, m: month, N/A: not available, ASA-PS: American Society of Anesthesiologists physical status, MILS: manual in-line stabilization.

**Table 2:** Summary of findings table for primary outcome.

**Patients:** pediatric patients who received tracheal intubation			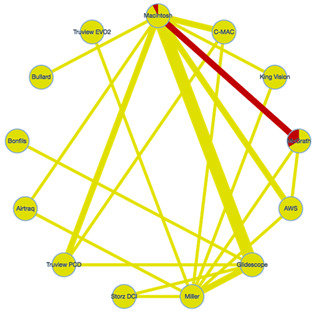	
**Interventions:** indirect laryngoscope, miller laryngoscope				
**Comparator (reference):** macintosh laryngoscope				
**Outcome:** failure of tracheal intubation at first attempt				
**Setting:** elective surgery				
**Total studies: 31 RCT** **Total Participants: 1930**	**Relative effect** **(95% CI)**	**Certainty of evidence**	**Reasons for** **downgrading**	**P score**
Airtraq (1 RCT; 34 participants)	0.27 (0.04–1.61)	⨁**◯◯◯** **Low**	Risk of bias and imprecision	0.90
Airway scope (2 RCT; 137 participants)	0.50 (0.16–1.51)	⨁**◯◯◯** **Low**	Risk of bias and imprecision	0.81
Bonfils (1 RCT; 84 participants)	1.08 (0.10–11.3)	⨁**◯◯◯** **Low**	Risk of bias and imprecision	0.57
Bullard (1 RCT; 40 participants)	19.0 (0.91–398.0)	⨁**◯◯◯** **Low**	Risk of bias and heterogeneity	0.08
C-MAC (3 RCT; 256 participants)	1.16 (0.25–5.45)	⨁**◯◯◯** **Low**	Risk of bias and imprecision	0.55
Glidescope (5 RCT; 408 participants)	1.52 (0.58–4.00)	⨁**◯◯◯** **Low**	Risk of bias and imprecision	0.45
King Vision (1 RCT; 78 participants)	4.23 (0.43–41.5)	⨁**◯◯◯** **Low**	Risk of bias and imprecision	0.23
Macintosh (13 RCT; 659 participants)	No estimable	**Reference comparator**	No estimable	0.60
McGrath (3 RCT; 347 participants)	0.55 (0.20–1.51)	⨁**◯◯◯** **Low**	Risk of bias and imprecision	0.79
Miller (8 RCT; 594 participants)	1.41 (0.48–4.18)	⨁**◯◯◯** **Low**	Risk of bias and imprecision	0.48
Storz DCI (1 RCT; 62 participants)	2.87 (0.52–15.7)	⨁**◯◯◯** **Low**	Risk of bias and imprecision	0.28
Truview EVO2 (1 RCT; 60 participants)	0.94 (0.09–10.2)	⨁**◯◯◯** **Low**	Risk of bias and imprecision	0.60
Truview PCD (2 RCT; 142 participants)	4.78 (1.11–20.6)	⨁**◯◯◯** **Low**	Risk of bias and imprecision	0.17

RCT: Randomized controlled trial, CI: Confidence interval.

## Data Availability

Not applicable.

## Supplementary Materials

The following supporting information can be downloaded at: https://www.mdpi.com/article/10.3390/children9091280/s1, Figure S1: Search strategy, Figure S2: Methodological domain of risks of bias, Figure S3: Meta-analysis flow chart, Figure S4: League table of the primary outcome, Figure S5: Results of inconsistency of the primary outcome, Figure S6: Results of funnel plot of the primary (A) and secondary outcome (B,C), Figure S7: League table of the secondary outcome (glottic visualization), Figure S8: Results of inconsistency of the secondary outcome (glottic visualization), Figure S9: Summary of findings table for the secondary outcome (glottic visualization), Figure S10: League table of the secondary outcome (intubation time), Figure S11: Results of inconsistency of the secondary outcome (intubation time), Figure S12: Summary of findings table for the secondary outcome (intubation time), Figure S13: Forest plot of the subgroup analysis, Figure S14: The results of subgroup analysis by P score ranking, Figure S15: The results of funnel plot of the subgroup analysis.
