# Supplementary material for: Effectiveness of Indirect and Direct Laryngoscopes in Pediatric Patients: A Systematic Review and Network Meta-Analysis
Source: Children (Basel). 2022 Aug 25;9(9):1280. doi: 10.3390/children9091280 (PMC9497385; doi:10.3390/children9091280)
Supplement: Supplementary file 1 [file children-09-01280-s001.zip › children-1864936-supplementary.pdf]

## Supplementary Materials

**Search strategy for Pubmed**

(("paediatrics"[All Fields] OR "pediatrics"[MeSH Terms] OR "pediatrics"[All Fields] OR "paediatric"[All Fields] OR "pediatric"[All Fields]) AND ("laryngoscope s"[All Fields] OR "laryngoscopes"[MeSH Terms] OR "laryngoscopes"[All Fields] OR "laryngoscope"[All Fields] OR "laryngoscopic"[All Fields] OR "laryngoscopical"[All Fields] OR "laryngoscopically"[All Fields]) AND ("intubate"[All Fields] OR "intubated"[All Fields] OR "intubates"[All Fields] OR "intubating"[All Fields] OR "intubation"[MeSH Terms] OR "intubation"[All Fields] OR "intubations"[All Fields] OR "intubator"[All Fields] OR "intubators"[All Fields])) AND (humans[Filter])

**Search terms for the Cochrane library**

#1 tracheal intubation

#2 intubation

#3 #1 or #2

#4 videolaryngoscope

#5 video laryngoscope

#6 videolaryngoscopes

#7 video laryngoscopes

#8 indirect laryngoscope

#9 #4 or #5 or #6 or #7 or #8

#10 paediatrics

#11 pediatrics

#12 paediatric

#13 pediatric

#14 child

#15 # 10 or #11 or #12 or #13 or #14

#16 #3 and #9 and #15

**Search strategy for EMBASE**

(tracheal intubation OR intubation)

AND (videolaryngoscope OR video laryngoscope OR videolaryngoscopes OR video laryngoscopes OR indirectlaryngoscope OR indirect laryngoscope)

AND (paediatrics OR pediatrics OR paediatric OR pediatric OR child)

**Figure S1.** Search strategy.

The risks of bias was estimated in the following methodological domains: sequence generation; allocation concealment; blinding of participants; incomplete outcome data; selective outcome reporting; and other potential threats to validity.

**Figure S2.** The methodological domain of risks of bias.

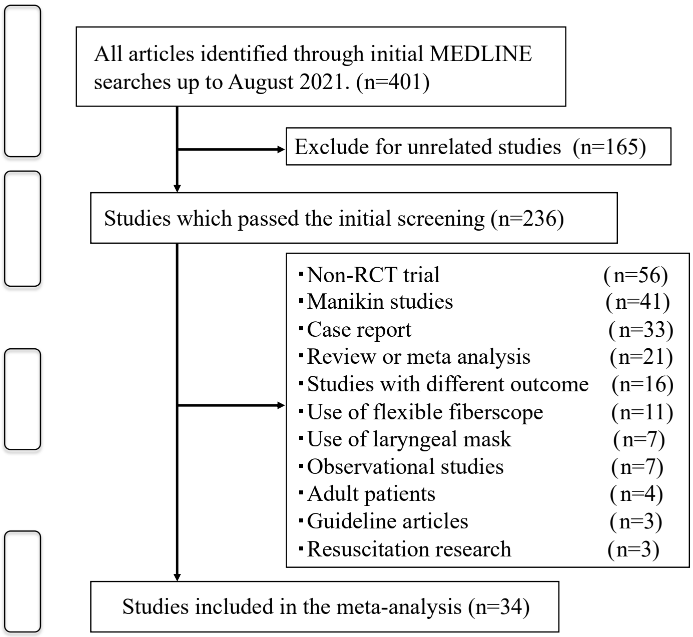


**Figure S3.** Meta-analysis flow chart. RCT, randomized controlled trial.

| | | | | | | | | | | | | |
|---|---|---|---|---|---|---|---|---|---|---|---|---|
| **Airtraq** | . | . | . | . | . | . | 0.25 (0.02; 2.84) | . | 0.20 (0.02; 2.27) | . | . | . |
| 0.53 (0.07; 4.03) | **AWS** | . | . | . | . | . | 0.32 (0.08; 1.31) | 1.38 (0.21; 9.05) | 0.64 (0.12; 3.53) | . | . | . |
| 0.25 (0.01; 4.35) | 0.46 (0.04; 5.82) | **Bonfils** | . | . | 0.71 (0.08; 6.06) | . | . | . | . | . | . | . |
| 0.01 (0.00; 0.48) | 0.03 (0.00; 0.67) | 0.06 (0.00; 2.66) | **Bullard** | . | . | . | 19.00 (0.91; 398.73) | . | . | . | . | . |
| 0.23 (0.02; 2.13) | 0.43 (0.07; 2.57) | 0.93 (0.06; 13.50) | 16.35 (0.54; 496.97) | **C-MAC** | . | . | 3.00 (0.27; 33.29) | . | 0.41 (0.06; 3.05) | . | . | 0.33 (0.04; 2.99) |
| 0.17 (0.03; 1.19) | 0.33 (0.08; 1.28) | 0.71 (0.08; 6.06) | 12.52 (0.51; 305.31) | 0.77 (0.15; 3.80) | **Glidescope** | . | 1.59 (0.51; 4.94) | . | 1.03 (0.27; 3.90) | 0.71 (0.14; 3.61) | . | 0.34 (0.05; 2.48) |
| 0.06 (0.00; 0.93) | 0.12 (0.01; 1.28) | 0.26 (0.01; 5.78) | 4.49 (0.10; 202.07) | 0.27 (0.02; 3.37) | 0.36 (0.04; 3.47) | **King Vision** | . | . | 3.00 (0.40; 22.37) | . | . | . |
| 0.27 (0.04; 1.61) | 0.50 (0.16; 1.51) | 1.08 (0.10; 11.33) | 19.00 (0.91; 398.73) | 1.16 (0.25; 5.45) | 1.52 (0.58; 4.00) | 4.23 (0.43; 41.50) | **Macintosh** | 1.75 (0.52; 5.85) | . | . | . | 0.14 (0.02; 0.98) |
| 0.49 (0.07; 3.58) | 0.91 (0.24; 3.40) | 1.98 (0.16; 24.38) | 34.76 (1.40; 861.22) | 2.13 (0.37; 12.37) | 2.78 (0.75; 10.33) | 7.73 (0.71; 83.71) | 1.83 (0.66; 5.07) | **McGrath** | 0.47 (0.08; 2.81) | . | . | . |
| 0.19 (0.03; 1.14) | 0.35 (0.10; 1.28) | 0.77 (0.07; 8.34) | 13.48 (0.53; 341.60) | 0.82 (0.18; 3.70) | 1.08 (0.38; 3.08) | 3.00 (0.40; 22.37) | 0.71 (0.24; 2.10) | 0.39 (0.11; 1.39) | **Miller** | 0.29 (0.04; 1.99) | 1.50 (0.18; 12.51) | . |
| 0.09 (0.01; 0.94) | 0.17 (0.03; 1.17) | 0.38 (0.03; 5.16) | 6.63 (0.20; 216.42) | 0.41 (0.05; 3.24) | 0.53 (0.12; 2.38) | 1.47 (0.11; 19.29) | 0.35 (0.06; 1.91) | 0.19 (0.03; 1.26) | 0.49 (0.10; 2.45) | **Storz DCI** | . | . |
| 0.28 (0.02; 4.57) | 0.53 (0.04; 6.35) | 1.15 (0.05; 28.00) | 20.23 (0.42; 965.62) | 1.24 (0.09; 16.61) | 1.62 (0.15; 17.24) | 4.50 (0.24; 83.55) | 1.06 (0.10; 11.54) | 0.58 (0.05; 6.92) | 1.50 (0.18; 12.51) | 3.05 (0.21; 43.62) | **Truview EVO2** | . |
| 0.06 (0.01; 0.52) | 0.10 (0.02; 0.61) | 0.23 (0.02; 3.04) | 3.98 (0.14; 116.29) | 0.24 (0.05; 1.28) | 0.32 (0.07; 1.38) | 0.88 (0.07; 11.48) | 0.21 (0.05; 0.90) | 0.11 (0.02; 0.64) | 0.29 (0.06; 1.45) | 0.60 (0.08; 4.61) | 0.20 (0.01; 2.79) | **Truview PCD** |

**Figure S4.** League table of the primary outcome.

| Comparison | AWS:Macintosh |
|---|---|
| Evidence: mixed | |
| NMA odds ratio: | 2.733(0.701,10.663) |
| Direct odds ratio: | 4.242(0.764,23.549) |
| Indirect odds ratio: | 1.289(0.137,12.118) |
| **Inconsistency measures** | |
| Ratio of odds ratios: | 3.291(0.196,55.263) |
| P value: | 0.408 |
| Incoherence judgment | No concerns |

| Comparison | AWS:McGrath |
|---|---|
| Evidence: mixed | |
| NMA odds ratio: | 1.030(0.199,5.329) |
| Direct odds ratio: | 0.696(0.069,7.052) |
| Indirect odds ratio: | 1.533(0.149,15.811) |
| **Inconsistency measures** | |
| Ratio of odds ratios: | 0.454(0.017,12.152) |
| P value: | 0.638 |
| Incoherence judgment | No concerns |

| Comparison | AWS:Miller |
|---|---|
| Evidence: mixed | |
| NMA odds ratio: | 3.415(0.697,16.720) |
| Direct odds ratio: | 1.696(0.192,14.989) |
| Indirect odds ratio: | 7.552(0.742,76.869) |
| **Inconsistency measures** | |
| Ratio of odds ratios: | 0.225(0.009,5.417) |
| P value: | 0.358 |
| Incoherence judgment | No concerns |

| Comparison | Airtraq:Macintosh |
|---|---|
| Evidence: mixed | |
| NMA odds ratio: | 4.776(0.586,38.951) |
| Direct odds ratio: | 4.923(0.282,85.841) |
| Indirect odds ratio: | 4.611(0.210,101.403) |
| **Inconsistency measures** | |
| Ratio of odds ratios: | 1.068(0.016,71.909) |
| P value: | 0.976 |
| Incoherence judgment | No concerns |

| Comparison | Airtraq:Miller |
|---|---|
| Evidence: mixed | |
| NMA odds ratio: | 5.968(0.740,48.139) |
| Direct odds ratio: | 5.800(0.360,93.513) |
| Indirect odds ratio: | 6.192(0.262,146.145) |
| **Inconsistency measures** | |
| Ratio of odds ratios: | 0.937(0.014,63.080) |
| P value: | 0.976 |
| Incoherence judgment | No concerns |

| Comparison | Bonfils:Glidescope |
|---|---|
| Evidence: direct | |
| Direct odds ratio: | 1.432(0.118,17.317) |
| **Inconsistency measures:** Not applicable | |
| Incoherence judgment | No concerns |

| Comparison | Bullard:Macintosh |
|---|---|
| Evidence: direct | |
| Direct odds ratio: | 0.030(0.001,0.869) |
| **Inconsistency measures:** Not applicable | |
| Incoherence judgment | No concerns |

| Comparison | C-MAC:Macintosh |
|---|---|
| Evidence: mixed | |
| NMA odds ratio: | 0.869(0.149,5.076) |
| Direct odds ratio: | 0.324(0.025,4.270) |
| Indirect odds ratio: | 2.074(0.184,23.331) |
| **Inconsistency measures** | |
| Ratio of odds ratios: | 0.156(0.005,5.367) |
| P value: | 0.304 |
| Incoherence judgment | No concerns |

| Comparison | C-MAC:Miller |
|---|---|
| Evidence: mixed | |
| NMA odds ratio: | 1.086(0.188,6.283) |
| Direct odds ratio: | 2.685(0.242,29.830) |
| Indirect odds ratio: | 0.389(0.030,5.054) |
| **Inconsistency measures** | |
| Ratio of odds ratios: | 6.902(0.205,232.595) |
| P value: | 0.282 |
| Incoherence judgment | No concerns |

| Comparison | C-MAC:Truview PCD |
|---|---|
| Evidence: mixed | |
| NMA odds ratio: | 4.565(0.661,31.526) |
| Direct odds ratio: | 3.193(0.254,40.149) |
| Indirect odds ratio: | 7.519(0.378,149.635) |
| **Inconsistency measures** | |
| Ratio of odds ratios: | 0.425(0.008,21.372) |
| P value: | 0.668 |
| Incoherence judgment | No concerns |

| Comparison | Glidescope:Macintosh |
|---|---|
| Evidence: mixed | |
| NMA odds ratio: | 0.670(0.213,2.107) |
| Direct odds ratio: | 0.605(0.158,2.316) |
| Indirect odds ratio: | 0.880(0.097,7.973) |
| **Inconsistency measures** | |
| Ratio of odds ratios: | 0.688(0.052,9.072) |
| P value: | 0.776 |
| Incoherence judgment | No concerns |

| Comparison | Glidescope:Miller |
|---|---|
| Evidence: mixed | |
| NMA odds ratio: | 0.837(0.235,2.985) |
| Direct odds ratio: | 0.957(0.185,4.951) |
| Indirect odds ratio: | 0.686(0.092,5.098) |
| **Inconsistency measures** | |
| Ratio of odds ratios: | 1.395(0.104,18.664) |
| P value: | 0.801 |
| Incoherence judgment | No concerns |

| Comparison | Glidescope:Storz DCI |
|---|---|
| **Evidence: mixed** | |
| NMA odds ratio: | 2.132(0.305,14.910) |
| Direct odds ratio: | 1.517(0.184,12.530) |
| Indirect odds ratio: | 14.322(0.097,2109.276) |
| **Inconsistency measures** | |
| Ratio of odds ratios: | 0.106(0.000,23.932) |
| P value: | 0.417 |
| Incoherence judgment | No concerns |

| Comparison | Glidescope:Truview PCD |
|---|---|
| **Evidence: mixed** | |
| NMA odds ratio: | 3.518(0.618,20.019) |
| Direct odds ratio: | 3.231(0.304,34.326) |
| Indirect odds ratio: | 3.891(0.299,50.694) |
| **Inconsistency measures** | |
| Ratio of odds ratios: | 0.830(0.025,27.205) |
| P value: | 0.917 |
| Incoherence judgment | No concerns |

| Comparison | King Vision:Macintosh |
|---|---|
| **Evidence: mixed** | |
| NMA odds ratio: | 0.350(0.036,3.414) |
| Direct odds ratio: | 1.000(0.014,72.879) |
| Indirect odds ratio: | 0.231(0.016,3.407) |
| **Inconsistency measures** | |
| Ratio of odds ratios: | 4.320(0.027,682.116) |
| P value: | 0.571 |
| Incoherence judgment | No concerns |

| Comparison | King Vision:Miller |
|---|---|
| **Evidence: mixed** | |
| NMA odds ratio: | 0.437(0.055,3.481) |
| Direct odds ratio: | 0.320(0.031,3.318) |
| Indirect odds ratio: | 1.381(0.016,122.940) |
| **Inconsistency measures** | |
| Ratio of odds ratios: | 0.231(0.001,36.558) |
| P value: | 0.571 |
| Incoherence judgment | No concerns |

| Comparison | Macintosh:McGrath |
|---|---|
| **Evidence: mixed** | |
| NMA odds ratio: | 0.377(0.102,1.397) |
| Direct odds ratio: | 0.382(0.078,1.869) |
| Indirect odds ratio: | 0.367(0.036,3.714) |
| **Inconsistency measures** | |
| Ratio of odds ratios: | 1.039(0.063,17.193) |
| P value: | 0.979 |
| Incoherence judgment | No concerns |

| Comparison | Macintosh:Truview PCD |
|---|---|
| **Evidence: mixed** | |
| NMA odds ratio: | 5.252(0.963,28.654) |
| Direct odds ratio: | 7.824(0.928,65.957) |
| Indirect odds ratio: | 2.637(0.160,43.480) |
| **Inconsistency measures** | |
| Ratio of odds ratios: | 2.967(0.088,100.354) |
| P value: | 0.545 |
| Incoherence judgment | No concerns |

| Comparison | McGrath:Miller |
|---|---|
| **Evidence: mixed** | |
| NMA odds ratio: | 3.314(0.681,16.137) |
| Direct odds ratio: | 2.435(0.257,23.081) |
| Indirect odds ratio: | 4.486(0.484,41.621) |
| **Inconsistency measures** | |
| Ratio of odds ratios: | 0.543(0.023,12.865) |
| P value: | 0.705 |
| Incoherence judgment | No concerns |

| Comparison | Miller:Storz DCI |
|---|---|
| **Evidence: mixed** | |
| NMA odds ratio: | 2.548(0.336,19.294) |
| Direct odds ratio: | 4.229(0.397,45.038) |
| Indirect odds ratio: | 0.636(0.013,31.874) |
| **Inconsistency measures** | |
| Ratio of odds ratios: | 6.649(0.069,644.258) |
| P value: | 0.417 |
| Incoherence judgment | No concerns |

| Comparison | Miller:Truview EVO2 |
|---|---|
| **Evidence: direct** | |
| Direct odds ratio: | 0.643(0.052,7.933) |
| **Inconsistency measures:** Not applicable | |
| Incoherence judgment | No concerns |

| Comparison | Airtraq:AWS |
|---|---|
| **Evidence: indirect** | |
| Indirect odds ratio: | 1.748(0.159,19.269) |
| **Inconsistency measures:** Not applicable | |
| Incoherence judgment | No concerns |

| Comparison | AWS:Bonfils |
|---|---|
| **Evidence: indirect** | |
| Indirect odds ratio: | 2.849(0.142,57.111) |
| **Inconsistency measures:** Not applicable | |
| Incoherence judgment | No concerns |

| Comparison | AWS:Bullard |
|---|---|
| **Evidence: indirect** | |
| Indirect odds ratio: | 92.573(2.415,3548.570) |
| **Inconsistency measures:** Not applicable | |
| Incoherence judgment | No concerns |

| Comparison | AWS:C-MAC |
|---|---|
| **Evidence: indirect** | |
| Indirect odds ratio: | 3.144(0.379,26.073) |
| **Inconsistency measures:** Not applicable | |
| Incoherence judgment | No concerns |

| Comparison | AWS:Glidescope |
|---|---|
| **Evidence: indirect** | |
| Indirect odds ratio: | 4.080(0.772,21.574) |
| **Inconsistency measures:** Not applicable | |
| Incoherence judgment | No concerns |

**Comparison** **AWS:King Vision**
**Evidence: indirect**
Indirect odds ratio: **7.813(0.635,96.130)**
**Inconsistency measures:** Not applicable
Incoherence judgment: No concerns

**Comparison** **AWS:Storz DCI**
**Evidence: indirect**
Indirect odds ratio: **8.700(0.782,96.805)**
**Inconsistency measures:** Not applicable
Incoherence judgment: No concerns

**Comparison** **AWS:Truview EVO2**
**Evidence: indirect**
Indirect odds ratio: **2.195(0.112,42.910)**
**Inconsistency measures:** Not applicable
Incoherence judgment: No concerns

**Comparison** **AWS:Truview PCD**
**Evidence: indirect**
Indirect odds ratio: **14.355(1.743,118.250)**
**Inconsistency measures:** Not applicable
Incoherence judgment: No concerns

**Comparison** **Airtraq:Bonfils**
**Evidence: indirect**
Indirect odds ratio: **4.980(0.174,142.451)**
**Inconsistency measures:** Not applicable
Incoherence judgment: No concerns

**Comparison** **Airtraq:Bullard**
**Evidence: indirect**
Indirect odds ratio: **161.790(3.021,8664.591)**
**Inconsistency measures:** Not applicable
Incoherence judgment: No concerns

**Comparison** **Airtraq:C-MAC**
**Evidence: indirect**
Indirect odds ratio: **5.495(0.416,72.574)**
**Inconsistency measures:** Not applicable
Incoherence judgment: No concerns

**Comparison** **Airtraq:Glidescope**
**Evidence: indirect**
Indirect odds ratio: **7.131(0.757,67.209)**
**Inconsistency measures:** Not applicable
Incoherence judgment: No concerns

**Comparison** **Airtraq:King Vision**
**Evidence: indirect**
Indirect odds ratio: **13.655(0.766,243.447)**
**Inconsistency measures:** Not applicable
Incoherence judgment: No concerns

**Comparison** **Airtraq:McGrath**
**Evidence: indirect**
Indirect odds ratio: **1.801(0.166,19.537)**
**Inconsistency measures:** Not applicable
Incoherence judgment: No concerns

**Comparison** **Bonfils:Truview EVO2**
**Evidence: indirect**
Indirect odds ratio: **0.770(0.018,33.119)**
**Inconsistency measures:** Not applicable
Incoherence judgment: No concerns

**Comparison** **Bonfils:Truview PCD**
**Evidence: indirect**
Indirect odds ratio: **5.037(0.241,105.225)**
**Inconsistency measures:** Not applicable
Incoherence judgment: No concerns

**Comparison** **Bullard:C-MAC**
**Evidence: indirect**
Indirect odds ratio: **0.034(0.001,1.542)**
**Inconsistency measures:** Not applicable
Incoherence judgment: No concerns

**Comparison** **Bullard:Glidescope**
**Evidence: indirect**
Indirect odds ratio: **0.044(0.001,1.568)**
**Inconsistency measures:** Not applicable
Incoherence judgment: No concerns

**Comparison** **Bullard:King Vision**
**Evidence: indirect**
Indirect odds ratio: **0.084(0.001,4.983)**
**Inconsistency measures:** Not applicable
Incoherence judgment: No concerns

**Comparison** **Bullard:McGrath**
**Evidence: indirect**
Indirect odds ratio: **0.011(0.000,0.419)**
**Inconsistency measures:** Not applicable
Incoherence judgment: No concerns

**Comparison** **Bullard:Miller**
**Evidence: indirect**
Indirect odds ratio: **0.037(0.001,1.372)**
**Inconsistency measures:** Not applicable
Incoherence judgment: No concerns

**Comparison** **Bullard:Storz DCI**
**Evidence: indirect**
Indirect odds ratio: **0.094(0.002,5.142)**
**Inconsistency measures:** Not applicable
Incoherence judgment: No concerns

**Comparison** **Bullard:Truview EVO2**
**Evidence: indirect**
Indirect odds ratio: **0.024(0.000,1.939)**
**Inconsistency measures:** Not applicable
Incoherence judgment: No concerns

**Comparison** **Bullard:Truview PCD**
**Evidence: indirect**
Indirect odds ratio: **0.155(0.004,6.824)**
**Inconsistency measures:** Not applicable
Incoherence judgment: No concerns

| Comparison | Evidence | Indirect odds ratio | Inconsistency measures | Incoherence judgment |
|---|---|---|---|---|
| Airtraq:Storz DCI | indirect | 15.205(0.916,252.447) | Not applicable | No concerns |
| Airtraq:Truview EVO2 | indirect | 3.837(0.146,100.635) | Not applicable | No concerns |
| Airtraq:Truview PCD | indirect | 25.088(1.854,339.441) | Not applicable | No concerns |
| Bonfils:Bullard | indirect | 32.486(0.417,2530.570) | Not applicable | No concerns |
| Bonfils:C-MAC | indirect | 1.103(0.049,24.896) | Not applicable | No concerns |
| Bonfils:King Vision | indirect | 2.742(0.090,83.747) | Not applicable | No concerns |
| Bonfils:Macintosh | indirect | 0.959(0.062,14.908) | Not applicable | No concerns |
| Bonfils:McGrath | indirect | 0.362(0.018,7.139) | Not applicable | No concerns |
| Bonfils:Miller | indirect | 1.198(0.073,19.674) | Not applicable | No concerns |
| Bonfils:Storz DCI | indirect | 3.053(0.129,72.082) | Not applicable | No concerns |
| C-MAC:Glidescope | indirect | 1.298(0.200,8.421) | Not applicable | No concerns |
| C-MAC:King Vision | indirect | 2.485(0.176,35.146) | Not applicable | No concerns |
| C-MAC:McGrath | indirect | 0.328(0.040,2.668) | Not applicable | No concerns |
| C-MAC:Storz DCI | indirect | 2.767(0.218,35.156) | Not applicable | No concerns |
| C-MAC:Truview EVO2 | indirect | 0.698(0.033,14.968) | Not applicable | No concerns |
| Glidescope:King Vision | indirect | 1.915(0.184,19.886) | Not applicable | No concerns |
| Glidescope:McGrath | indirect | 0.253(0.049,1.300) | Not applicable | No concerns |
| Glidescope:Truview EVO2 | indirect | 0.538(0.032,8.993) | Not applicable | No concerns |
| King Vision:McGrath | indirect | 0.132(0.011,1.608) | Not applicable | No concerns |
| King Vision:Storz DCI | indirect | 1.113(0.064,19.304) | Not applicable | No concerns |

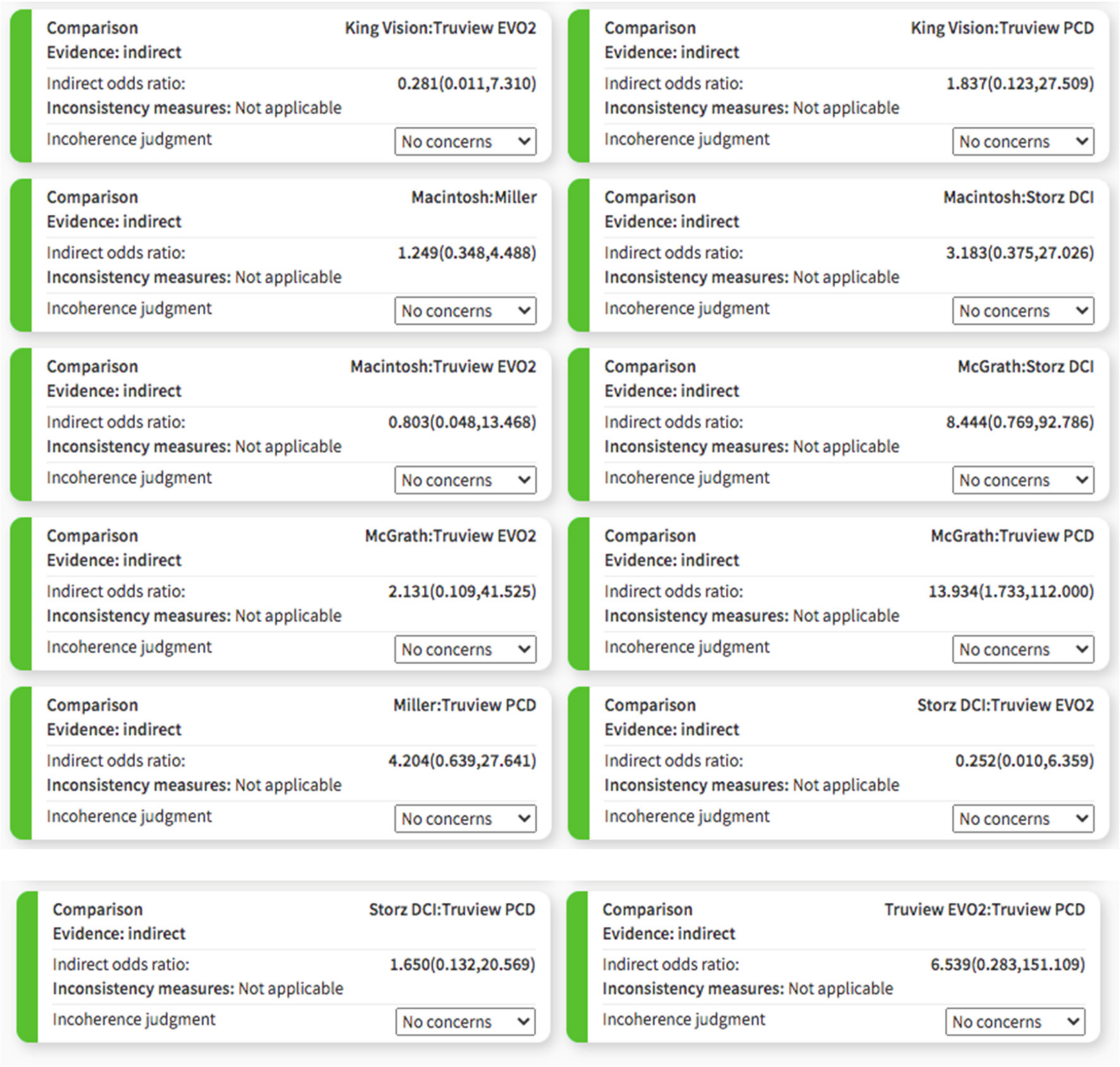

Comparison King Vision:Truview EVO2
Evidence: indirect
Indirect odds ratio: 0.281(0.011,7.310)
Inconsistency measures: Not applicable
Incoherence judgment No concerns

Comparison King Vision:Truview PCD
Evidence: indirect
Indirect odds ratio: 1.837(0.123,27.509)
Inconsistency measures: Not applicable
Incoherence judgment No concerns

Comparison Macintosh:Miller
Evidence: indirect
Indirect odds ratio: 1.249(0.348,4.488)
Inconsistency measures: Not applicable
Incoherence judgment No concerns

Comparison Macintosh:Storz DCI
Evidence: indirect
Indirect odds ratio: 3.183(0.375,27.026)
Inconsistency measures: Not applicable
Incoherence judgment No concerns

Comparison Macintosh:Truview EVO2
Evidence: indirect
Indirect odds ratio: 0.803(0.048,13.468)
Inconsistency measures: Not applicable
Incoherence judgment No concerns

Comparison McGrath:Storz DCI
Evidence: indirect
Indirect odds ratio: 8.444(0.769,92.786)
Inconsistency measures: Not applicable
Incoherence judgment No concerns

Comparison McGrath:Truview EVO2
Evidence: indirect
Indirect odds ratio: 2.131(0.109,41.525)
Inconsistency measures: Not applicable
Incoherence judgment No concerns

Comparison McGrath:Truview PCD
Evidence: indirect
Indirect odds ratio: 13.934(1.733,112.000)
Inconsistency measures: Not applicable
Incoherence judgment No concerns

Comparison Miller:Truview PCD
Evidence: indirect
Indirect odds ratio: 4.204(0.639,27.641)
Inconsistency measures: Not applicable
Incoherence judgment No concerns

Comparison Storz DCI:Truview EVO2
Evidence: indirect
Indirect odds ratio: 0.252(0.010,6.359)
Inconsistency measures: Not applicable
Incoherence judgment No concerns

Comparison Storz DCI:Truview PCD
Evidence: indirect
Indirect odds ratio: 1.650(0.132,20.569)
Inconsistency measures: Not applicable
Incoherence judgment No concerns

Comparison Truview EVO2:Truview PCD
Evidence: indirect
Indirect odds ratio: 6.539(0.283,151.109)
Inconsistency measures: Not applicable
Incoherence judgment No concerns

**Figure S5.** Results of inconsistency of the primary outcome.

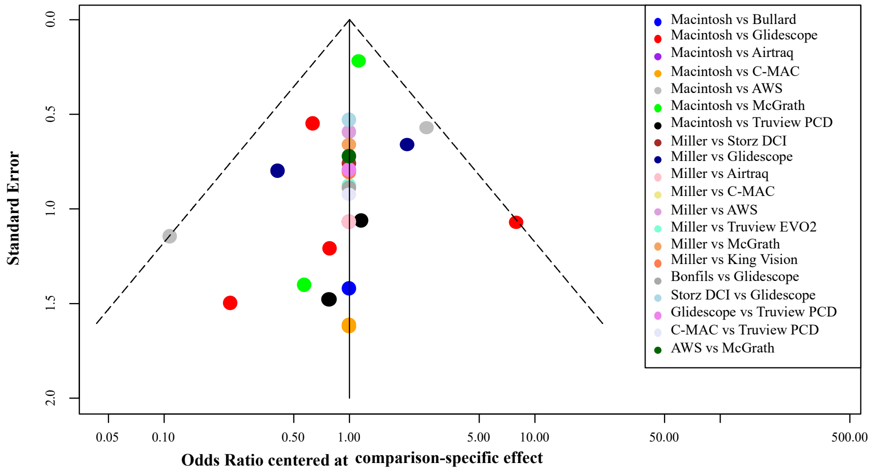


**(A)**

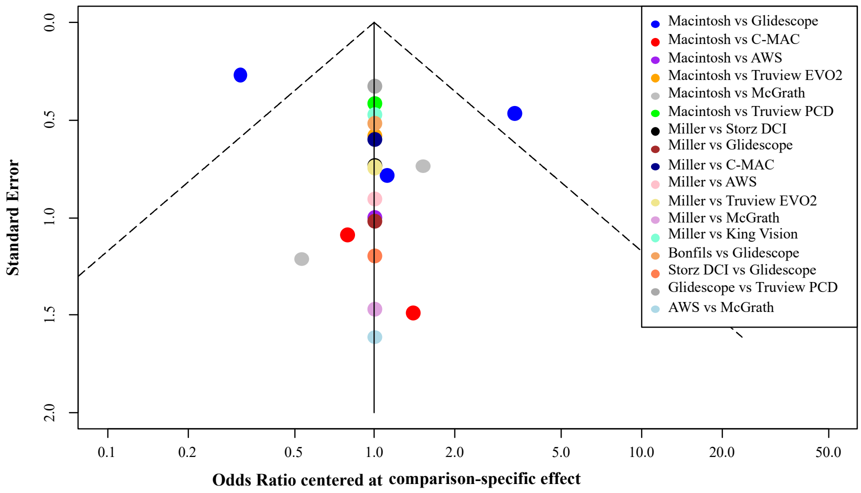


**(B)**

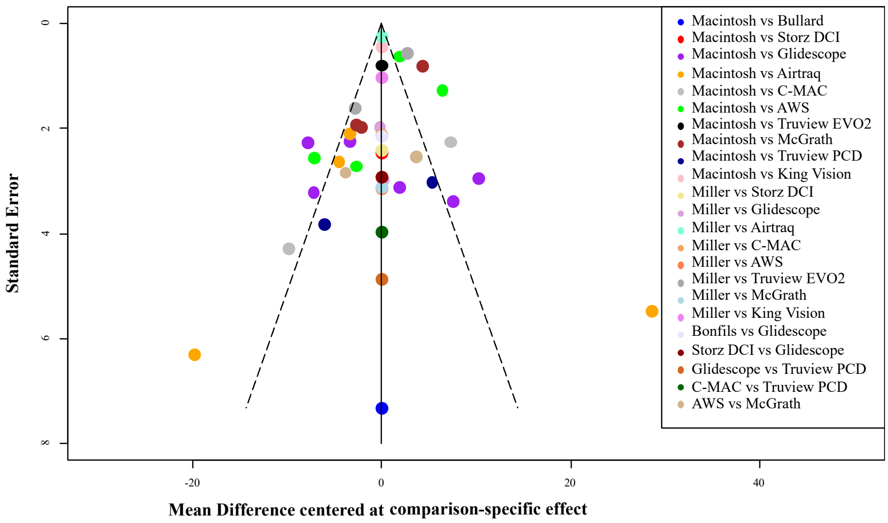


**(C)**

**Figure S6.** Results of funnel plot of the primary (**A**) and secondary outcome (**B, C**),.

| | | | | | | | | | | |
|---|---|---|---|---|---|---|---|---|---|---|
| **AWS** | | | | | 0.03 (0.00; 0.59) | 3.10 (0.07; 145.21) | 0.32 (0.02; 5.42) | | | |
| 0.15 (0.00; 4.42) | **Bonfils** | | 1.25 (0.11; 13.93) | | | | | | | |
| 0.51 (0.04; 7.09) | 3.48 (0.14; 89.62) | **C-MAC** | | | 0.20 (0.02; 2.07) | | 0.28 (0.02; 3.39) | | | |
| 0.18 (0.02; 2.02) | 1.25 (0.11; 13.93) | 0.36 (0.04; 3.14) | **Glidescope** | | 1.11 (0.27; 4.48) | | 0.10 (0.01; 1.94) | 0.50 (0.02; 12.42) | | 4.86 (0.50; 47.66) |
| 0.22 (0.01; 5.57) | 1.49 (0.03; 70.10) | 0.43 (0.02; 9.04) | 1.20 (0.06; 24.04) | **King Vision** | | | 0.46 (0.04; 4.99) | | | |
| 0.14 (0.02; 1.16) | 0.95 (0.06; 14.66) | 0.27 (0.04; 1.76) | 0.76 (0.21; 2.78) | 0.64 (0.04; 11.29) | **Macintosh** | 3.64 (0.47; 27.90) | | | 4.00 (0.34; 47.27) | 1.38 (0.13; 14.24) |
| 0.67 (0.05; 8.54) | 4.59 (0.18; 117.88) | 1.32 (0.11; 15.94) | 3.68 (0.42; 32.27) | 3.08 (0.12; 77.98) | 4.84 (0.80; 29.18) | **McGrath** | 0.10 (0.00; 3.88) | | | |
| 0.10 (0.01; 0.91) | 0.69 (0.03; 14.20) | 0.20 (0.03; 1.33) | 0.55 (0.09; 3.43) | 0.46 (0.04; 4.99) | 0.73 (0.14; 3.65) | 0.15 (0.02; 1.33) | **Miller** | 5.00 (0.36; 68.66) | 4.00 (0.62; 25.72) | |
| 0.30 (0.01; 7.63) | 2.05 (0.05; 80.78) | 0.59 (0.03; 12.45) | 1.64 (0.10; 26.25) | 1.38 (0.04; 44.11) | 2.16 (0.14; 34.54) | 0.45 (0.02; 10.73) | 2.98 (0.24; 37.12) | **Storz DCI** | | |
| 0.45 (0.04; 5.59) | 3.09 (0.13; 74.96) | 0.89 (0.09; 8.66) | 2.48 (0.31; 19.93) | 2.07 (0.12; 36.43) | 3.26 (0.53; 19.90) | 0.67 (0.06; 7.52) | 4.49 (0.91; 22.19) | 1.51 (0.08; 27.57) | **Truview EVO2** | |
| 0.43 (0.02; 8.34) | 2.92 (0.12; 71.88) | 0.84 (0.05; 13.63) | 2.34 (0.28; 19.27) | 1.96 (0.06; 65.35) | 3.08 (0.37; 25.86) | 0.64 (0.04; 10.11) | 4.24 (0.32; 55.81) | 1.42 (0.05; 41.20) | 0.94 (0.06; 14.55) | **Truview PCD** |

**Figure S7.** League table of the secondary outcome (glottic visualization).

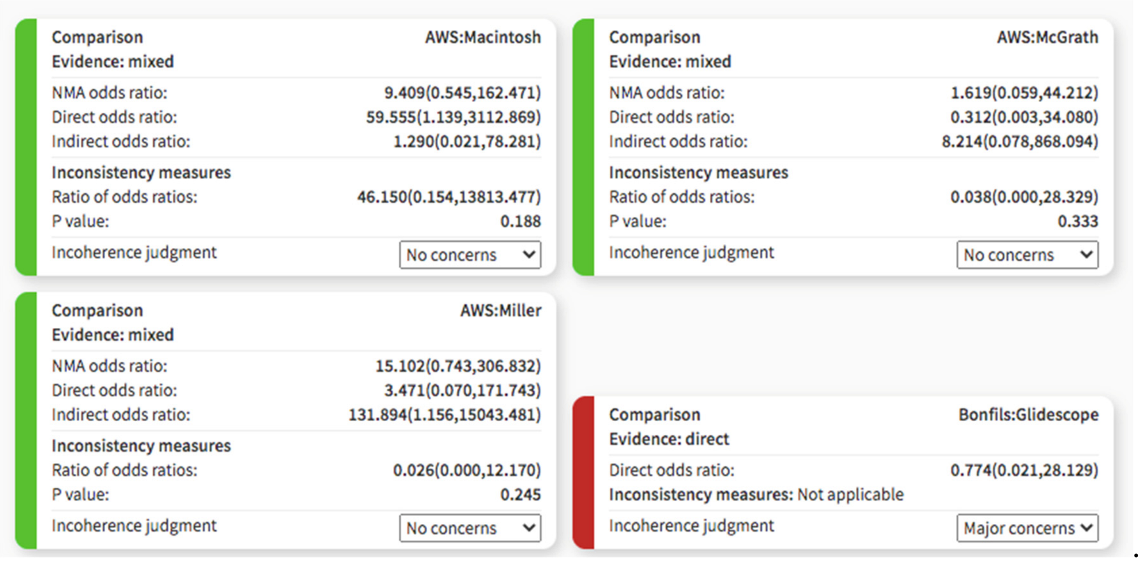

.

| Comparison | C-MAC:Macintosh |
|---|---|
| Evidence: mixed | |
| NMA odds ratio: | 4.436(0.360,54.615) |
| Direct odds ratio: | 5.733(0.276,118.914) |
| Indirect odds ratio: | 2.537(0.029,223.096) |
| **Inconsistency measures** | |
| Ratio of odds ratios: | 2.260(0.010,503.911) |
| P value: | 0.767 |
| Incoherence judgment | No concerns |

| Comparison | C-MAC:Miller |
|---|---|
| Evidence: mixed | |
| NMA odds ratio: | 7.120(0.481,105.436) |
| Direct odds ratio: | 4.889(0.124,192.116) |
| Indirect odds ratio: | 11.050(0.209,585.286) |
| **Inconsistency measures** | |
| Ratio of odds ratios: | 0.442(0.002,98.642) |
| P value: | 0.767 |
| Incoherence judgment | No concerns |

| Comparison | Glidescope:Macintosh |
|---|---|
| Evidence: mixed | |
| NMA odds ratio: | 1.051(0.154,7.195) |
| Direct odds ratio: | 0.584(0.072,4.778) |
| Indirect odds ratio: | 21.911(0.184,2606.856) |
| **Inconsistency measures** | |
| Ratio of odds ratios: | 0.027(0.000,4.935) |
| P value: | 0.174 |
| Incoherence judgment | No concerns |

| Comparison | Glidescope:Miller |
|---|---|
| Evidence: mixed | |
| NMA odds ratio: | 1.687(0.129,22.003) |
| Direct odds ratio: | 14.286(0.260,786.270) |
| Indirect odds ratio: | 0.381(0.013,10.807) |
| **Inconsistency measures** | |
| Ratio of odds ratios: | 37.487(0.203,6935.442) |
| P value: | 0.174 |
| Incoherence judgment | No concerns |

| Comparison | Glidescope:Storz DCI |
|---|---|
| Evidence: mixed | |
| NMA odds ratio: | 0.567(0.013,24.150) |
| Direct odds ratio: | 2.069(0.031,136.497) |
| Indirect odds ratio: | 0.003(0.000,13.763) |
| **Inconsistency measures** | |
| Ratio of odds ratios: | 688.489(0.056,8431624.365) |
| P value: | 0.174 |
| Incoherence judgment | No concerns |

| Comparison | Glidescope:Truview PCD |
|---|---|
| Evidence: mixed | |
| NMA odds ratio: | 0.160(0.006,4.086) |
| Direct odds ratio: | 0.034(0.001,1.231) |
| Indirect odds ratio: | 155.369(0.081,299269.565) |
| **Inconsistency measures** | |
| Ratio of odds ratios: | 0.000(0.000,0.948) |
| P value: | 0.049 |
| Incoherence judgment | No concerns |

.

| Comparison | King Vision:Miller |
|---|---|
| Evidence: direct | |
| Direct odds ratio: | 2.341(0.068,80.875) |
| **Inconsistency measures:** Not applicable | |
| Incoherence judgment | Major concerns |

| Comparison | Macintosh:McGrath |
|---|---|
| Evidence: mixed | |
| NMA odds ratio: | 0.172(0.016,1.899) |
| Direct odds ratio: | 0.250(0.015,4.050) |
| Indirect odds ratio: | 0.059(0.001,6.678) |
| **Inconsistency measures** | |
| Ratio of odds ratios: | 4.232(0.017,1024.336) |
| P value: | 0.607 |
| Incoherence judgment | No concerns |

| Comparison | Macintosh:Truview EVO2 |
|---|---|
| Evidence: mixed | |
| NMA odds ratio: | 0.238(0.017,3.273) |
| Direct odds ratio: | 0.148(0.004,5.901) |
| Indirect odds ratio: | 0.387(0.009,16.092) |
| **Inconsistency measures** | |
| Ratio of odds ratios: | 0.381(0.002,72.125) |
| P value: | 0.719 |
| Incoherence judgment | No concerns |

| Comparison | Macintosh:Truview PCD |
|---|---|
| Evidence: mixed | |
| NMA odds ratio: | 0.152(0.006,3.838) |
| Direct odds ratio: | 0.668(0.019,23.171) |
| Indirect odds ratio: | 0.000(0.000,0.293) |
| **Inconsistency measures** | |
| Ratio of odds ratios: | 5484.055(1.056,28485336.384) |
| P value: | 0.049 |
| Incoherence judgment | Major concerns |

| Comparison | McGrath:Miller |
|---|---|
| Evidence: mixed | |
| NMA odds ratio: | 9.325(0.502,173.157) |
| Direct odds ratio: | 11.117(0.122,1010.499) |
| Indirect odds ratio: | 8.211(0.177,380.201) |
| **Inconsistency measures** | |
| Ratio of odds ratios: | 1.354(0.004,504.214) |
| P value: | 0.920 |
| Incoherence judgment | No concerns |

| Comparison | Miller:Storz DCI |
|---|---|
| Evidence: mixed | |
| NMA odds ratio: | 0.336(0.010,11.824) |
| Direct odds ratio: | 0.145(0.003,6.228) |
| Indirect odds ratio: | 476.515(0.008,29793475.960) |
| **Inconsistency measures** | |
| Ratio of odds ratios: | 0.000(0.000,35.431) |
| P value: | 0.174 |
| Incoherence judgment | No concerns |

.

**Comparison** — **Miller:Truview EVO2**
**Evidence: mixed**
NMA odds ratio: **0.148(0.015,1.478)**
Direct odds ratio: **0.191(0.013,2.757)**
Indirect odds ratio: **0.073(0.001,6.608)**
**Inconsistency measures**
Ratio of odds ratios: **2.623(0.014,496.111)**
P value: **0.719**
Incoherence judgment: No concerns

**Comparison** — **AWS:Bonfils**
**Evidence: indirect**
Indirect odds ratio: **11.571(0.088,1526.603)**
**Inconsistency measures:** Not applicable
Incoherence judgment: Major concerns

**Comparison** — **AWS:C-MAC**
**Evidence: indirect**
Indirect odds ratio: **2.121(0.060,75.286)**
**Inconsistency measures:** Not applicable
Incoherence judgment: Major concerns

**Comparison** — **AWS:Glidescope**
**Evidence: indirect**
Indirect odds ratio: **8.950(0.328,243.886)**
**Inconsistency measures:** Not applicable
Incoherence judgment: Major concerns

**Comparison** — **AWS:King Vision**
**Evidence: indirect**
Indirect odds ratio: **6.451(0.062,674.317)**
**Inconsistency measures:** Not applicable
Incoherence judgment: Major concerns

**Comparison** — **AWS:Storz DCI**
**Evidence: indirect**
Indirect odds ratio: **5.075(0.059,438.298)**
**Inconsistency measures:** Not applicable
Incoherence judgment: Major concerns

**Comparison** — **AWS:Truview EVO2**
**Evidence: indirect**
Indirect odds ratio: **2.240(0.067,74.426)**
**Inconsistency measures:** Not applicable
Incoherence judgment: Major concerns

**Comparison** — **AWS:Truview PCD**
**Evidence: indirect**
Indirect odds ratio: **1.433(0.020,100.816)**
**Inconsistency measures:** Not applicable
Incoherence judgment: Major concerns

**Comparison** — **Bonfils:C-MAC**
**Evidence: indirect**
Indirect odds ratio: **0.183(0.002,20.017)**
**Inconsistency measures:** Not applicable
Incoherence judgment: Major concerns

**Comparison** — **Bonfils:King Vision**
**Evidence: indirect**
Indirect odds ratio: **0.557(0.002,160.405)**
**Inconsistency measures:** Not applicable
Incoherence judgment: Major concerns

**Comparison** — **Bonfils:Macintosh**
**Evidence: indirect**
Indirect odds ratio: **0.813(0.014,47.904)**
**Inconsistency measures:** Not applicable
Incoherence judgment: Major concerns

**Comparison** — **Bonfils:McGrath**
**Evidence: indirect**
Indirect odds ratio: **0.140(0.001,15.044)**
**Inconsistency measures:** Not applicable
Incoherence judgment: Major concerns

**Comparison** — **Bonfils:Miller**
**Evidence: indirect**
Indirect odds ratio: **1.305(0.016,108.115)**
**Inconsistency measures:** Not applicable
Incoherence judgment: Major concerns

**Comparison** — **Bonfils:Storz DCI**
**Evidence: indirect**
Indirect odds ratio: **0.439(0.002,79.115)**
**Inconsistency measures:** Not applicable
Incoherence judgment: Major concerns

**Comparison** — **Bonfils:Truview EVO2**
**Evidence: indirect**
Indirect odds ratio: **0.194(0.002,21.181)**
**Inconsistency measures:** Not applicable
Incoherence judgment: Major concerns

**Comparison** — **Bonfils:Truview PCD**
**Evidence: indirect**
Indirect odds ratio: **0.124(0.001,15.635)**
**Inconsistency measures:** Not applicable
Incoherence judgment: Major concerns

**Comparison** — **C-MAC:Glidescope**
**Evidence: indirect**
Indirect odds ratio: **4.220(0.206,86.349)**
**Inconsistency measures:** Not applicable
Incoherence judgment: Major concerns

**Comparison** — **C-MAC:King Vision**
**Evidence: indirect**
Indirect odds ratio: **3.041(0.035,260.708)**
**Inconsistency measures:** Not applicable
Incoherence judgment: Major concerns

| Comparison | Evidence | Indirect odds ratio | Inconsistency measures | Incoherence judgment |
|---|---|---|---|---|
| C-MAC:McGrath | indirect | 0.764(0.027,21.452) | Not applicable | Major concerns |
| C-MAC:Storz DCI | indirect | 2.393(0.034,167.922) | Not applicable | Major concerns |
| C-MAC:Truview EVO2 | indirect | 1.056(0.042,26.832) | Not applicable | Major concerns |
| C-MAC:Truview PCD | indirect | 0.676(0.012,38.199) | Not applicable | Major concerns |
| Glidescope:King Vision | indirect | 0.721(0.009,57.277) | Not applicable | Major concerns |
| Glidescope:McGrath | indirect | 0.181(0.009,3.612) | Not applicable | Major concerns |
| Glidescope:Truview EVO2 | indirect | 0.250(0.012,5.137) | Not applicable | Major concerns |
| King Vision:Macintosh | indirect | 1.459(0.022,97.251) | Not applicable | Major concerns |
| King Vision:McGrath | indirect | 0.251(0.003,24.769) | Not applicable | Major concerns |
| King Vision:Storz DCI | indirect | 0.787(0.005,119.426) | Not applicable | Major concerns |

.

| Comparison | Evidence | Indirect odds ratio | Inconsistency measures | Incoherence judgment |
|---|---|---|---|---|
| King Vision:Truview EVO2 | indirect | 0.347(0.005,23.691) | Not applicable | Major concerns |
| King Vision:Truview PCD | indirect | 0.222(0.001,40.029) | Not applicable | Major concerns |
| Macintosh:Miller | indirect | 1.605(0.168,15.322) | Not applicable | Major concerns |
| Macintosh:Storz DCI | indirect | 0.539(0.012,24.690) | Not applicable | Major concerns |
| McGrath:Storz DCI | indirect | 3.134(0.041,238.913) | Not applicable | Major concerns |
| McGrath:Truview EVO2 | indirect | 1.383(0.049,38.784) | Not applicable | Major concerns |
| McGrath:Truview PCD | indirect | 0.885(0.016,47.904) | Not applicable | Major concerns |
| Miller:Truview PCD | indirect | 0.095(0.002,4.236) | Not applicable | Major concerns |
| Storz DCI:Truview EVO2 | indirect | 0.441(0.007,26.851) | Not applicable | Major concerns |
| Storz DCI:Truview PCD | indirect | 0.282(0.002,34.012) | Not applicable | Major concerns |

.

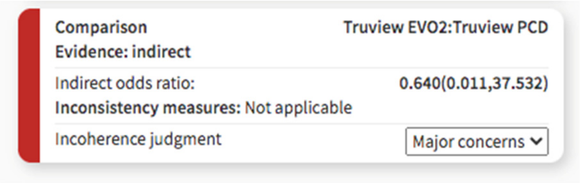


**Figure S8.** Results of inconsistency of the secondary outcome (glottic visualization).

**Patients**: pediatric patients who received tracheal intubation

**Interventions**: indirect laryngoscope, direct laryngoscope

**Comparator (reference)**: macintosh laryngoscope

**Outcome**: glottic visualization

**Setting**: elective surgery

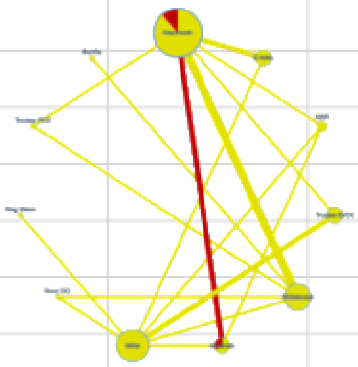

| **Total studies: 16 RCT**<br>**Total Participants: 1459** | **Relative effect (95% CI)** | **Certainty of evidence** | **Reasons for downgrading** | **P score** |
|---|---|---|---|---|
| Airway scope<br>(2 RCT; 195 participants) | 0.14 (0.02-1.16) | ⊕▯ ▯ ▯ **Low** | Risk of bias and imprecision | 0.81 |
| Bonfils<br>(1 RCT; 91 participants) | 0.095 (0.06-14.7) | ⊕▯ ▯ ▯ **Low** | Risk of bias and imprecision | 0.34 |
| C-MAC<br>(2 RCT; 116 participants) | 0.27 (0.04-1.76) | ⊕▯ ▯ ▯ **Low** | Risk of bias and imprecision | 0.66 |
| Glidescope<br>(3 RCT; 226 participants) | 0.76 (0.21-2.78) | ⊕▯ ▯ ▯ **Low** | Risk of bias and imprecision | 0.36 |
| King Vision<br>(1 RCT; 100 participants) | 0.64 (0.04-11.3) | ⊕▯ ▯ ▯ **Low** | Risk of bias and imprecision | 0.43 |
| Macintosh<br>(9 RCT; 384 participants) | No estimable | **Reference comparator** | No estimable | 0.26 |
| McGrath<br>(2 RCT; 216 participants) | 0.21 (0.03-1.24) | ⊕▯ ▯ ▯ **Low** | Risk of bias, imprecision and heterogeneity | 0.73 |
| Miller<br>(5 RCT; 489 participants) | 1.38 (0.27-6.93) | ⊕▯ ▯ ▯ **Low** | Risk of bias and imprecision | 0.18 |
| Storz DCI<br>(1 RCT; 62 participants) | 0.46 (0.03-7.39) | ⊕▯ ▯ ▯ **Low** | Risk of bias and imprecision | 0.51 |
| Truview EVO2<br>(1 RCT; 50 participants) | 0.31 (0.05-1.87) | ⊕▯ ▯ ▯ **Low** | Risk of bias and imprecision | 0.17 |
| Truview PCD<br>(2 RCT; 156 participants) | 0.32 (0.04-2.73) | ⊕▯ ▯ ▯ **Low** | Risk of bias and imprecision | 0.61 |

RCT: Randomized controlled trial, CI: Confidence interval

**Figure S9.** Summary of findings table for the secondary outcome (glottic visualization).

| | | | | | | | | | | | | |
|---|---|---|---|---|---|---|---|---|---|---|---|---|
| **Airtraq** | | | | | | | 1.2 ( -5.6; 7.9) | | -3.6 (-14.6; 7.4) | | | |
| -1.4 ( -9.1; 6.4) | **AWS** | | | | | | 2.4 ( -3.4; 8.2) | 0.3 ( -8.3; 9.0) | -4.0 (-16.6; 8.6) | | | |
| 5.8 ( -8.0; 19.6) | 7.2 ( -6.3; 20.8) | **Bonfils** | | | -12.2 (-24.0; -0.4) | | | | | | | |
| -37.0 (-56.0; -18.0) | -35.6 (-54.4; -16.8) | -42.8 (-64.8; -20.8) | **Bullard** | | | | 37.0 ( 18.9; 55.1) | | | | | |
| 9.4 ( 0.3; 18.5) | 10.8 ( 2.0; 19.6) | 3.6 (-10.7; 17.9) | 46.4 ( 26.9; 65.9) | **C-MAC** | | | -13.0 (-22.0; -4.0) | | -6.1 (-17.9; 5.7) | | | -12.7 (-26.2; 0.8) |
| -6.4 (-13.5; 0.8) | -5.0 (-11.7; 1.7) | -12.2 (-24.0; -0.4) | 30.6 ( 12.0; 49.2) | -15.8 (-24.0; -7.6) | **Glidescope** | | 8.8 ( 3.8; 13.8) | | 0.9 ( -7.6; 9.4) | -8.9 (-21.3; 3.5) | | -5.0 (-19.6; 9.6) |
| -3.6 (-13.4; 6.2) | -2.2 (-11.8; 7.3) | -9.4 (-24.3; 5.4) | 33.4 ( 13.5; 53.3) | -13.0 (-23.6; -2.5) | 2.8 ( -6.3; 11.8) | **King Vision** | 0.9 (-10.2; 11.9) | | 5.9 ( -5.3; 17.1) | | | |
| 0.0 ( -5.9; 6.0) | 1.4 ( -3.8; 6.6) | -5.8 (-18.4; 6.8) | 37.0 ( 18.9; 55.1) | -9.4 (-16.7; -2.1) | 6.4 ( 2.0; 10.9) | 3.6 ( -4.6; 11.9) | **Macintosh** | -3.4 (-10.0; 3.3) | | -6.5 (-18.5; 5.5) | 5.9 ( -5.2; 17.0) | -14.9 (-24.1; -5.8) |
| -2.2 (-10.2; 5.9) | -0.8 ( -7.4; 5.8) | -8.0 (-21.8; 5.7) | 34.8 ( 15.8; 53.7) | -11.6 (-20.7; -2.6) | 4.2 ( -2.9; 11.2) | 1.4 ( -8.4; 11.2) | -2.2 ( -7.9; 3.4) | **McGrath** | -0.5 (-13.1; 12.1) | | | |
| -0.5 ( -7.4; 6.3) | 0.9 ( -5.9; 7.6) | -6.4 (-19.5; 6.8) | 36.5 ( 17.7; 55.2) | -10.0 (-17.7; -2.2) | 5.8 ( 0.1; 11.6) | 3.1 ( -5.2; 11.3) | -0.6 ( -5.6; 4.5) | 1.7 ( -5.4; 8.7) | **Miller** | -10.0 (-22.0; 2.0) | -4.6 (-12.5; 3.4) | |
| -9.9 (-19.9; 0.0) | -8.6 (-18.2; 1.1) | -15.8 (-30.4; -1.2) | 27.0 ( 7.2; 46.9) | -19.4 (-30.1; -8.7) | -3.6 (-12.2; 5.0) | -6.4 (-17.6; 4.9) | -10.0 (-18.2; -1.7) | -7.8 (-17.6; 2.1) | -9.4 (-18.1; -0.7) | **Storz DCI** | | |
| -1.4 (-10.2; 7.5) | 0.0 ( -8.6; 8.7) | -7.2 (-21.4; 7.1) | 35.6 ( 16.1; 55.1) | -10.8 (-20.5; -1.1) | 5.0 ( -3.0; 13.1) | 2.2 ( -8.0; 12.4) | -1.4 ( -8.7; 5.9) | 0.8 ( -8.1; 9.8) | -0.8 ( -7.5; 5.9) | 8.6 ( -1.8; 19.0) | **Truview EVO2** | |
| -11.4 (-21.6; -1.2) | -10.0 (-19.9; -0.2) | -17.2 (-32.1; -2.4) | 25.6 ( 5.6; 45.5) | -20.8 (-30.7; -11.0) | -5.0 (-14.1; 4.1) | -7.8 (-19.5; 3.8) | -11.4 (-19.9; -3.0) | -9.2 (-19.3; 0.9) | -10.9 (-20.4; -1.4) | -1.5 (-13.1; 10.2) | -10.1 (-21.0; 0.9) | **Truview PCD** |

**Figure S10.** League table of the secondary outcome (intubation time).

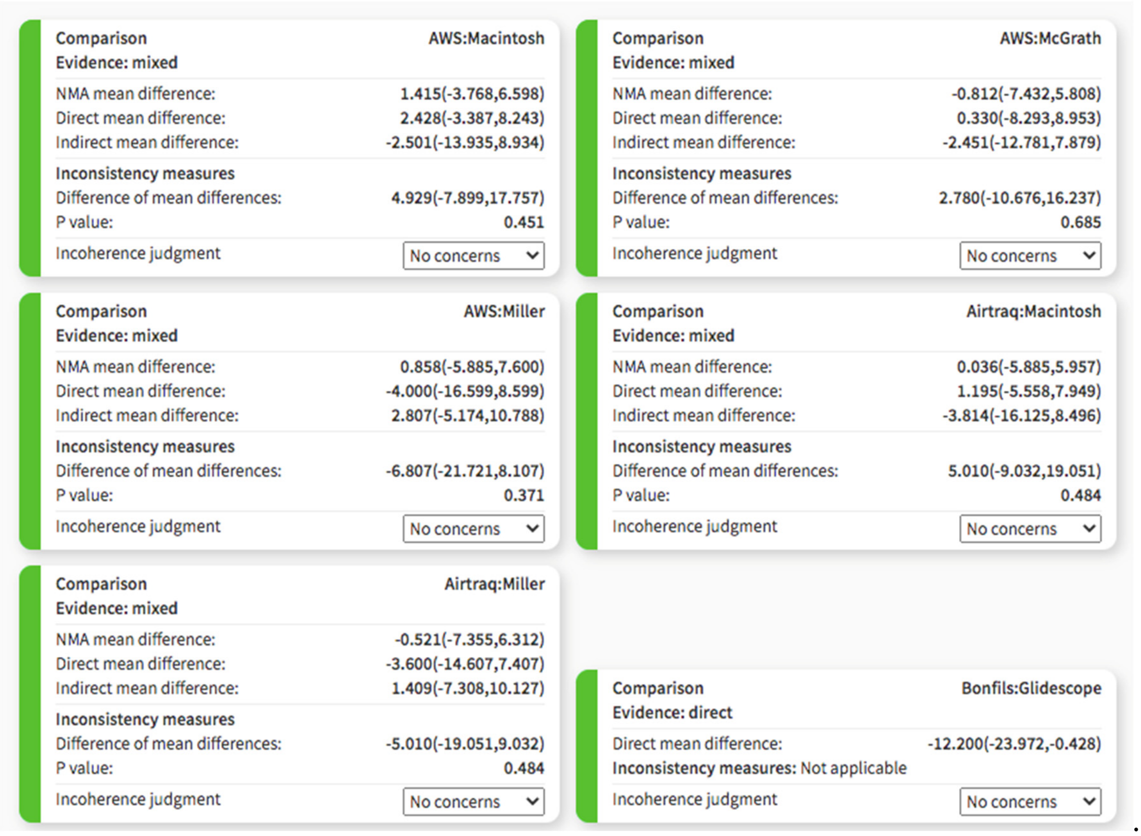

| Comparison | Evidence | NMA mean difference | Direct mean difference | Indirect mean difference | Inconsistency measures: Difference of mean differences | P value | Incoherence judgment |
|---|---|---|---|---|---|---|---|
| Bullard:Macintosh | direct | | 37.020(18.938,55.102) | | Not applicable | | No concerns |
| C-MAC:Macintosh | mixed | -9.396(-16.662,-2.131) | -12.989(-21.991,-3.987) | -2.679(-14.988,9.630) | -10.310(-25.560,4.939) | 0.185 | No concerns |
| C-MAC:Miller | mixed | -9.954(-17.686,-2.222) | -6.100(-17.864,5.664) | -12.885(-23.143,-2.626) | 6.785(-8.824,22.394) | 0.394 | No concerns |
| C-MAC:Truview PCD | mixed | -20.845(-30.714,-10.976) | -12.700(-26.180,0.780) | -30.253(-44.741,-15.765) | 17.553(-2.236,37.342) | 0.082 | Major concerns |
| Glidescope:Macintosh | mixed | 6.406(1.952,10.859) | 8.799(3.756,13.842) | -2.068(-11.557,7.421) | 10.867(0.121,21.613) | 0.048 | Major concerns |
| Glidescope:Miller | mixed | 5.848(0.085,11.611) | 0.913(-7.601,9.427) | 10.022(2.192,17.852) | -9.109(-20.676,2.458) | 0.123 | No concerns |
| Glidescope:Storz DCI | mixed | -3.578(-12.194,5.038) | -8.900(-21.301,3.501) | 1.389(-10.592,13.369) | -10.289(-27.531,6.954) | 0.242 | No concerns |
| Glidescope:Truview PCD | mixed | -5.043(-14.141,4.056) | -5.000(-19.564,9.564) | -5.070(-16.722,6.583) | 0.070(-18.582,18.721) | 0.994 | No concerns |
| King Vision:Macintosh | mixed | 3.625(-4.616,11.866) | 0.870(-10.159,11.899) | 7.107(-5.293,19.508) | -6.237(-22.833,10.358) | 0.461 | No concerns |
| King Vision:Miller | mixed | 3.067(-5.196,11.330) | 5.900(-5.284,17.084) | -0.338(-12.598,11.924) | 6.237(-10.358,22.833) | 0.461 | No concerns |
| Macintosh:McGrath | mixed | -2.227(-7.896,3.442) | -3.357(-9.972,3.258) | 0.897(-10.104,11.898) | -4.254(-17.090,8.583) | 0.516 | No concerns |
| Macintosh:Storz DCI | mixed | -9.984(-18.249,-1.719) | -6.500(-18.503,5.503) | -13.125(-24.521,-1.728) | 6.625(-9.927,23.177) | 0.433 | No concerns |

| Comparison | Macintosh:Truview EVO2 |
|---|---|
| **Evidence: mixed** | |
| NMA mean difference: | -1.389(-8.670,5.892) |
| Direct mean difference: | 5.900(-5.207,17.007) |
| Indirect mean difference: | -6.883(-16.525,2.760) |
| **Inconsistency measures** | |
| Difference of mean differences: | 12.783(-1.926,27.491) |
| P value: | 0.088 |
| Incoherence judgment | No concerns |

| Comparison | Macintosh:Truview PCD |
|---|---|
| **Evidence: mixed** | |
| NMA mean difference: | -11.448(-19.883,-3.014) |
| Direct mean difference: | -14.944(-24.052,-5.835) |
| Indirect mean difference: | 9.594(-12.754,31.943) |
| **Inconsistency measures** | |
| Difference of mean differences: | -24.538(-48.671,-0.405) |
| P value: | 0.046 |
| Incoherence judgment | Major concerns |

| Comparison | McGrath:Miller |
|---|---|
| **Evidence: mixed** | |
| NMA mean difference: | 1.670(-5.374,8.713) |
| Direct mean difference: | -0.500(-13.079,12.079) |
| Indirect mean difference: | 2.660(-5.840,11.162) |
| **Inconsistency measures** | |
| Difference of mean differences: | -3.160(-18.342,12.021) |
| P value: | 0.683 |
| Incoherence judgment | No concerns |

| Comparison | Miller:Storz DCI |
|---|---|
| **Evidence: mixed** | |
| NMA mean difference: | -9.426(-18.109,-0.744) |
| Direct mean difference: | -10.000(-21.965,1.966) |
| Indirect mean difference: | -8.788(-21.407,3.831) |
| **Inconsistency measures** | |
| Difference of mean differences: | -1.212(-18.602,16.178) |
| P value: | 0.891 |
| Incoherence judgment | No concerns |

| Comparison | Miller:Truview EVO2 |
|---|---|
| **Evidence: mixed** | |
| NMA mean difference: | -0.831(-7.519,5.856) |
| Direct mean difference: | -4.564(-12.512,3.384) |
| Indirect mean difference: | 8.219(-4.157,20.595) |
| **Inconsistency measures** | |
| Difference of mean differences: | -12.783(-27.491,1.926) |
| P value: | 0.088 |
| Incoherence judgment | No concerns |

| Comparison | Airtraq:AWS |
|---|---|
| **Evidence: indirect** | |
| Indirect mean difference: | -1.379(-9.123,6.366) |
| **Inconsistency measures:** Not applicable | |
| Incoherence judgment | No concerns |

.

| Comparison | AWS:Bonfils |
|---|---|
| **Evidence: indirect** | |
| Indirect mean difference: | 7.210(-6.340,20.759) |
| **Inconsistency measures:** Not applicable | |
| Incoherence judgment | No concerns |

| Comparison | AWS:Bullard |
|---|---|
| **Evidence: indirect** | |
| Indirect mean difference: | -35.605(-54.415,-16.795) |
| **Inconsistency measures:** Not applicable | |
| Incoherence judgment | No concerns |

| Comparison | AWS:C-MAC |
|---|---|
| **Evidence: indirect** | |
| Indirect mean difference: | 10.812(2.033,19.591) |
| **Inconsistency measures:** Not applicable | |
| Incoherence judgment | No concerns |

| Comparison | AWS:Glidescope |
|---|---|
| **Evidence: indirect** | |
| Indirect mean difference: | -4.990(-11.698,1.717) |
| **Inconsistency measures:** Not applicable | |
| Incoherence judgment | No concerns |

| Comparison | AWS:King Vision |
|---|---|
| **Evidence: indirect** | |
| Indirect mean difference: | -2.210(-11.764,7.344) |
| **Inconsistency measures:** Not applicable | |
| Incoherence judgment | No concerns |

| Comparison | AWS:Storz DCI |
|---|---|
| **Evidence: indirect** | |
| Indirect mean difference: | -8.569(-18.192,1.054) |
| **Inconsistency measures:** Not applicable | |
| Incoherence judgment | No concerns |

| Comparison | AWS:Truview EVO2 |
|---|---|
| **Evidence: indirect** | |
| Indirect mean difference: | 0.026(-8.645,8.697) |
| **Inconsistency measures:** Not applicable | |
| Incoherence judgment | No concerns |

| Comparison | AWS:Truview PCD |
|---|---|
| **Evidence: indirect** | |
| Indirect mean difference: | -10.033(-19.886,-0.180) |
| **Inconsistency measures:** Not applicable | |
| Incoherence judgment | No concerns |

| Comparison | Airtraq:Bonfils |
|---|---|
| **Evidence: indirect** | |
| Indirect mean difference: | 5.831(-7.957,19.619) |
| **Inconsistency measures:** Not applicable | |
| Incoherence judgment | No concerns |

| Comparison | Airtraq:Bullard |
|---|---|
| **Evidence: indirect** | |
| Indirect mean difference: | -36.984(-56.010,-17.957) |
| **Inconsistency measures:** Not applicable | |
| Incoherence judgment | No concerns |

.

**Comparison** **Airtraq:C-MAC**
**Evidence: indirect**
Indirect mean difference: **9.433(0.337,18.529)**
**Inconsistency measures:** Not applicable
Incoherence judgment: No concerns

**Comparison** **Airtraq:Glidescope**
**Evidence: indirect**
Indirect mean difference: **-6.369(-13.547,0.808)**
**Inconsistency measures:** Not applicable
Incoherence judgment: No concerns

**Comparison** **Airtraq:King Vision**
**Evidence: indirect**
Indirect mean difference: **-3.589(-13.389,6.212)**
**Inconsistency measures:** Not applicable
Incoherence judgment: No concerns

**Comparison** **Airtraq:McGrath**
**Evidence: indirect**
Indirect mean difference: **-2.191(-10.250,5.868)**
**Inconsistency measures:** Not applicable
Incoherence judgment: No concerns

**Comparison** **Airtraq:Storz DCI**
**Evidence: indirect**
Indirect mean difference: **-9.948(-19.861,-0.034)**
**Inconsistency measures:** Not applicable
Incoherence judgment: No concerns

**Comparison** **Airtraq:Truview EVO2**
**Evidence: indirect**
Indirect mean difference: **-1.353(-10.228,7.523)**
**Inconsistency measures:** Not applicable
Incoherence judgment: No concerns

**Comparison** **Airtraq:Truview PCD**
**Evidence: indirect**
Indirect mean difference: **-11.412(-21.629,-1.195)**
**Inconsistency measures:** Not applicable
Incoherence judgment: No concerns

**Comparison** **Bonfils:Bullard**
**Evidence: indirect**
Indirect mean difference: **-42.814(-64.845,-20.783)**
**Inconsistency measures:** Not applicable
Incoherence judgment: No concerns

**Comparison** **Bonfils:C-MAC**
**Evidence: indirect**
Indirect mean difference: **3.602(-10.737,17.941)**
**Inconsistency measures:** Not applicable
Incoherence judgment: No concerns

**Comparison** **Bonfils:King Vision**
**Evidence: indirect**
Indirect mean difference: **-9.419(-24.261,5.422)**
**Inconsistency measures:** Not applicable
Incoherence judgment: No concerns

**Comparison** **Bonfils:Macintosh**
**Evidence: indirect**
Indirect mean difference: **-5.794(-18.381,6.792)**
**Inconsistency measures:** Not applicable
Incoherence judgment: No concerns

**Comparison** **Bonfils:McGrath**
**Evidence: indirect**
Indirect mean difference: **-8.022(-21.754,5.711)**
**Inconsistency measures:** Not applicable
Incoherence judgment: No concerns

**Comparison** **Bonfils:Miller**
**Evidence: indirect**
Indirect mean difference: **-6.352(-19.459,6.755)**
**Inconsistency measures:** Not applicable
Incoherence judgment: No concerns

**Comparison** **Bonfils:Storz DCI**
**Evidence: indirect**
Indirect mean difference: **-15.778(-30.367,-1.190)**
**Inconsistency measures:** Not applicable
Incoherence judgment: No concerns

**Comparison** **Bonfils:Truview EVO2**
**Evidence: indirect**
Indirect mean difference: **-7.183(-21.441,7.075)**
**Inconsistency measures:** Not applicable
Incoherence judgment: No concerns

**Comparison** **Bonfils:Truview PCD**
**Evidence: indirect**
Indirect mean difference: **-17.243(-32.121,-2.364)**
**Inconsistency measures:** Not applicable
Incoherence judgment: No concerns

**Comparison** **Bullard:C-MAC**
**Evidence: indirect**
Indirect mean difference: **46.416(26.929,65.904)**
**Inconsistency measures:** Not applicable
Incoherence judgment: No concerns

**Comparison** **Bullard:Glidescope**
**Evidence: indirect**
Indirect mean difference: **30.614(11.992,49.236)**
**Inconsistency measures:** Not applicable
Incoherence judgment: No concerns

**Comparison** **Bullard:King Vision**
**Evidence: indirect**
Indirect mean difference: **33.395(13.524,53.266)**
**Inconsistency measures:** Not applicable
Incoherence judgment: No concerns

**Comparison** **Bullard:McGrath**
**Evidence: indirect**
Indirect mean difference: **34.793(15.843,53.742)**
**Inconsistency measures:** Not applicable
Incoherence judgment: No concerns

Comparison Bullard:Miller
**Evidence: indirect**
Indirect mean difference: **36.462(17.683,55.241)**
**Inconsistency measures:** Not applicable
Incoherence judgment: No concerns

Comparison Bullard:Storz DCI
**Evidence: indirect**
Indirect mean difference: **27.036(7.155,46.917)**
**Inconsistency measures:** Not applicable
Incoherence judgment: No concerns

Comparison Bullard:Truview EVO2
**Evidence: indirect**
Indirect mean difference: **35.631(16.138,55.124)**
**Inconsistency measures:** Not applicable
Incoherence judgment: No concerns

Comparison Bullard:Truview PCD
**Evidence: indirect**
Indirect mean difference: **25.572(5.619,45.524)**
**Inconsistency measures:** Not applicable
Incoherence judgment: No concerns

Comparison C-MAC:Glidescope
**Evidence: indirect**
Indirect mean difference: **-15.802(-23.989,-7.616)**
**Inconsistency measures:** Not applicable
Incoherence judgment: No concerns

Comparison C-MAC:King Vision
**Evidence: indirect**
Indirect mean difference: **-13.021(-23.580,-2.463)**
**Inconsistency measures:** Not applicable
Incoherence judgment: No concerns

Comparison C-MAC:McGrath
**Evidence: indirect**
Indirect mean difference: **-11.624(-20.676,-2.572)**
**Inconsistency measures:** Not applicable
Incoherence judgment: No concerns

Comparison C-MAC:Storz DCI
**Evidence: indirect**
Indirect mean difference: **-19.380(-30.059,-8.702)**
**Inconsistency measures:** Not applicable
Incoherence judgment: No concerns

Comparison C-MAC:Truview EVO2
**Evidence: indirect**
Indirect mean difference: **-10.786(-20.452,-1.119)**
**Inconsistency measures:** Not applicable
Incoherence judgment: No concerns

Comparison Glidescope:King Vision
**Evidence: indirect**
Indirect mean difference: **2.781(-6.257,11.818)**
**Inconsistency measures:** Not applicable
Incoherence judgment: No concerns

Comparison Glidescope:McGrath
**Evidence: indirect**
Indirect mean difference: **4.178(-2.893,11.249)**
**Inconsistency measures:** Not applicable
Incoherence judgment: No concerns

Comparison Glidescope:Truview EVO2
**Evidence: indirect**
Indirect mean difference: **5.017(-3.027,13.061)**
**Inconsistency measures:** Not applicable
Incoherence judgment: No concerns

Comparison King Vision:McGrath
**Evidence: indirect**
Indirect mean difference: **1.398(-8.400,11.195)**
**Inconsistency measures:** Not applicable
Incoherence judgment: No concerns

Comparison King Vision:Storz DCI
**Evidence: indirect**
Indirect mean difference: **-6.359(-17.630,4.912)**
**Inconsistency measures:** Not applicable
Incoherence judgment: No concerns

Comparison King Vision:Truview EVO2
**Evidence: indirect**
Indirect mean difference: **2.236(-7.971,12.443)**
**Inconsistency measures:** Not applicable
Incoherence judgment: No concerns

Comparison King Vision:Truview PCD
**Evidence: indirect**
Indirect mean difference: **-7.823(-19.476,3.829)**
**Inconsistency measures:** Not applicable
Incoherence judgment: No concerns

Comparison Macintosh:Miller
**Evidence: indirect**
Indirect mean difference: **-0.558(-5.627,4.512)**
**Inconsistency measures:** Not applicable
Incoherence judgment: No concerns

Comparison McGrath:Storz DCI
**Evidence: indirect**
Indirect mean difference: **-7.757(-17.629,2.116)**
**Inconsistency measures:** Not applicable
Incoherence judgment: No concerns

Comparison McGrath:Truview EVO2
**Evidence: indirect**
Indirect mean difference: **0.838(-8.090,9.767)**
**Inconsistency measures:** Not applicable
Incoherence judgment: No concerns

Comparison McGrath:Truview PCD
**Evidence: indirect**
Indirect mean difference: **-9.221(-19.331,0.889)**
**Inconsistency measures:** Not applicable
Incoherence judgment: No concerns

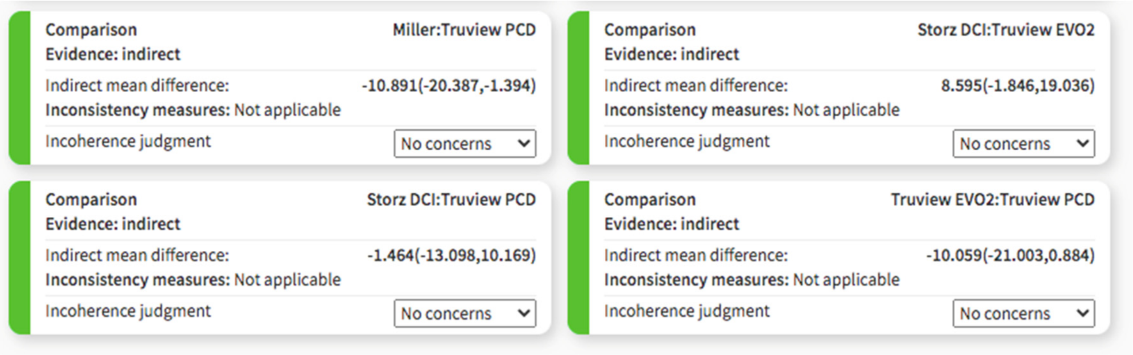


**Figure S11.** Results of inconsistency of the secondary outcome (intubation time).

**Patients**: pediatric patients who received tracheal intubation

**Interventions**: indirect laryngoscope, miller laryngoscope

**Comparator (reference)**: macintosh laryngoscope

**Outcome**: intubation time

**Setting**: elective surgery

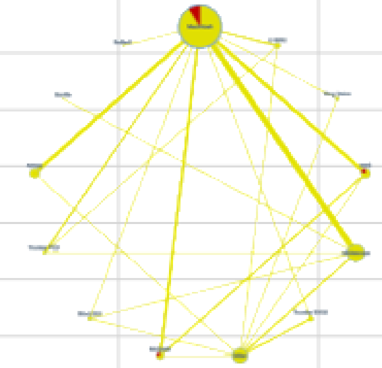

| Total studies: 31 RCT<br>Total Participants: 2599 | Mean diffence<br>(95% CI) | Certainty of evidence | Reasons for downgrading | P score |
|---|---|---|---|---|
| Airtraq<br>(1 RCT; 34 participants) | 0.56 (-5.88 to 5.96) | ⊕▯ ▯ ▯<br>Low | Risk of bias and imprecision | 0.66 |
| Airway scope<br>(2 RCT; 216 participants) | 1.42 (-3.77 to 6.60) | ⊕▯ ▯ ▯<br>Low | Risk of bias and imprecision | 0.57 |
| Bonfils<br>(1 RCT; 44 participants) | -5.79 (-18.4-6.79) | ⊕▯ ▯ ▯<br>Low | Risk of bias and imprecision | 0.85 |
| Bullard<br>(1 RCT; 40 participants) | 37.0 (18.9 to 55.1) | ???⊕⊕▯ ▯<br>Moderate | Risk of bias | 0.001 |
| C-MAC<br>(2 RCT; 131 participants) | -9.40 (-16.7 to -2.13) | ⊕▯ ▯ ▯<br>Low | Risk of bias and heterogeneity | 0.97 |
| Glidescope<br>(7 RCT; 671 participants) | 6.41 (1.95-10.9) | ⊕▯ ▯ ▯<br>Low | Risk of bias and imprecision | 0.28 |
| King Vision<br>(1 RCT; 78 participants) | 3.63 (-4.62 to 11.9) | ⊕▯ ▯ ▯<br>Low | Risk of bias and imprecision | 0.44 |
| Macintosh<br>(24 RCT; 1052 participants) | No estimable | Reference comparator | No estimable | 0.68 |
| McGrath<br>(3 RCT; 287 participants) | 2.23 (-3.44 to 7.90) | ⊕▯ ▯ ▯<br>Low | Risk of bias and imprecision | 0.52 |
| Miller<br>(6 RCT; 577 participants) | 0.56 (-4.51-5.63) | ⊕▯ ▯ ▯<br>Low | Risk of bias and imprecision | 0.63 |
| Storz DCI<br>(1 RCT; 60 participants) | 9.98 (1.72 to 18.3) | ⊕▯ ▯ ▯<br>Low | Risk of bias and heterogeneity | 0.18 |
| Truview EVO2<br>(3 RCT; 170 participants) | 1.39 (-5.89 to 8.67) | ⊕▯ ▯ ▯<br>Low | Risk of bias and imprecision | 0.57 |
| Truview PCD<br>(4 RCT; 380 participants) | 11.5 (3.01 to 19.9) | ⊕▯ ▯ ▯<br>Low | Risk of bias and heterogeneity | 0.15 |

RCT: Randomized controlled trial, CI:Confidence interval

**Figure S12.** Summary of findings table for the secondary outcome (intubation time).

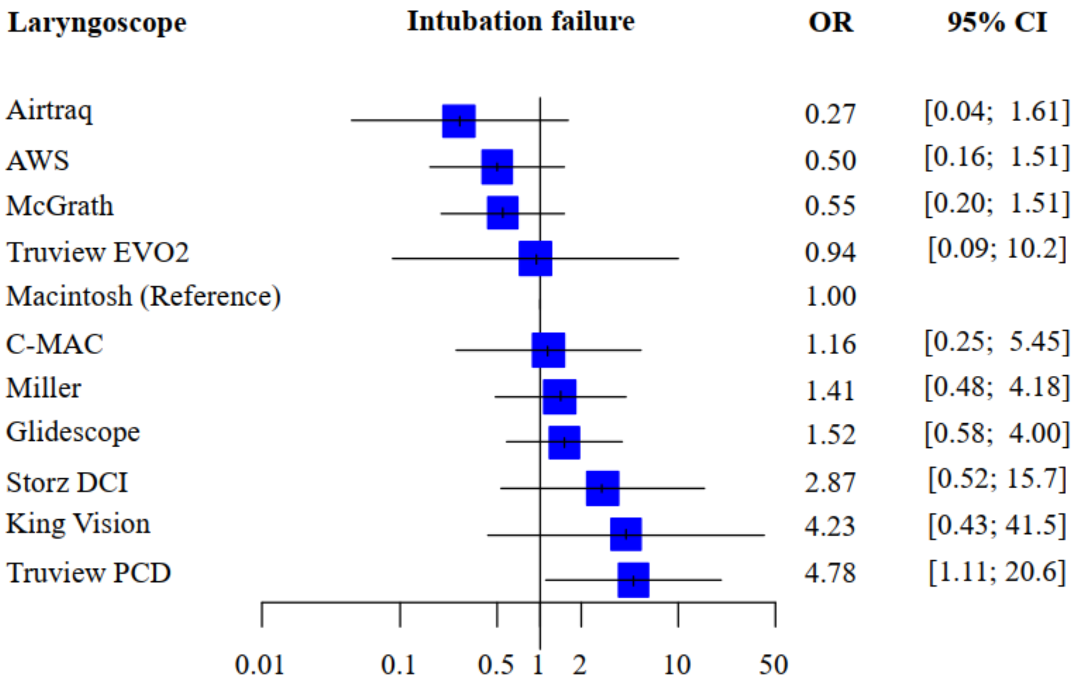


**(A)**

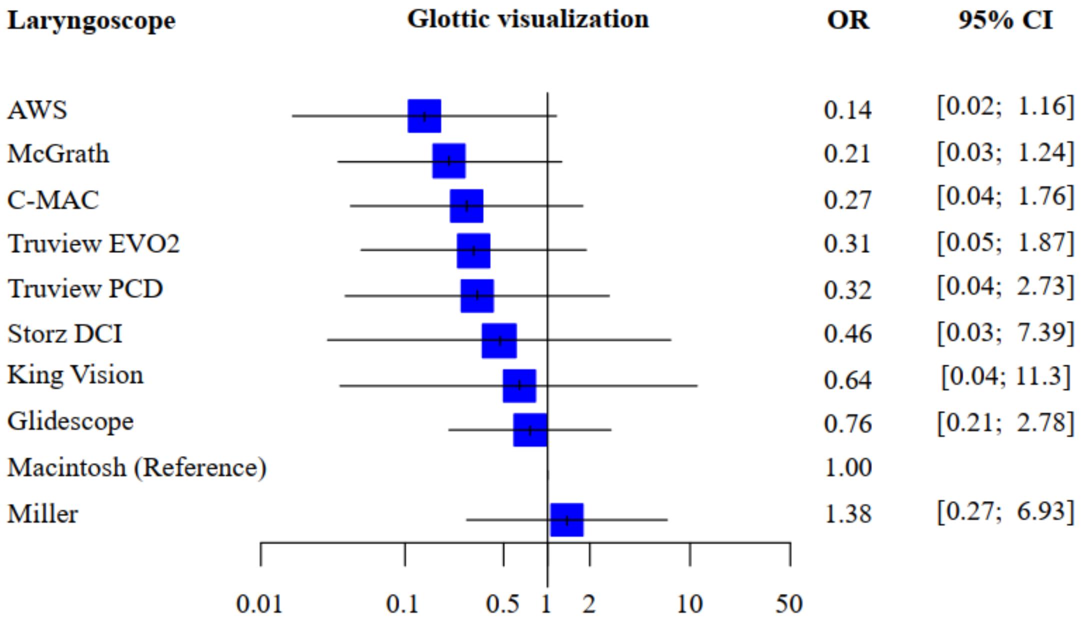


**(B)**

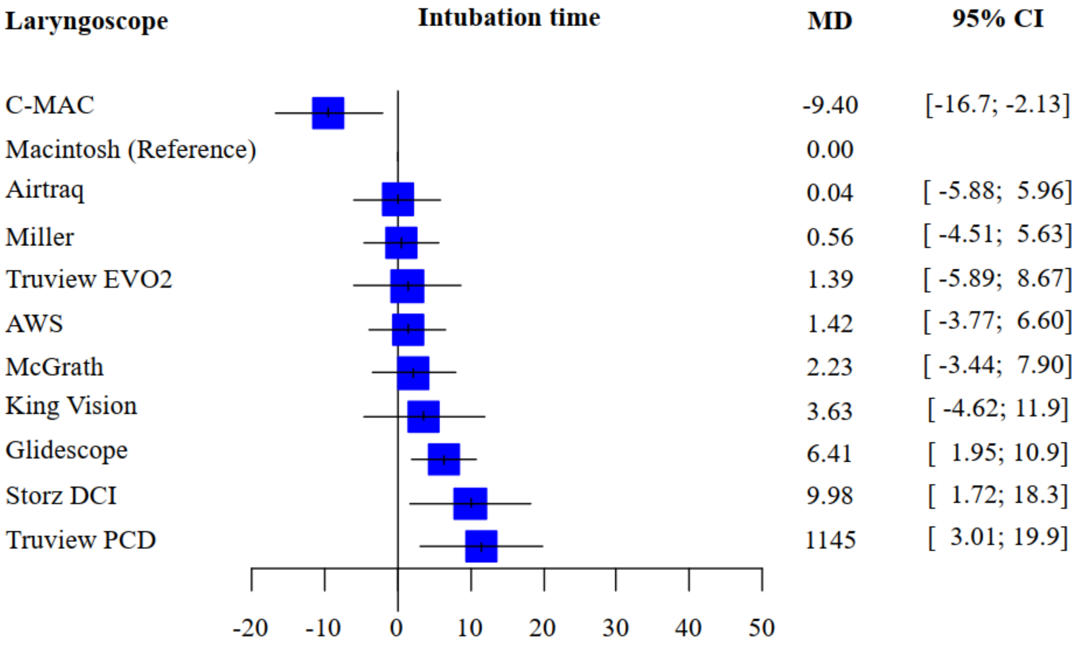


**(C)**

**Figure S13.** Forest plot of the subgroup analysis. (**A**:Forest plot of the intubation failure of tracheal intubation using the indirect laryngoscope compared with the Macintosh laryngoscope. **B**:Forest plot of glottic visualization with the indirect laryngoscope compared with the Macintosh laryngo-scope, Cormack–Lehane grade 1 and 2 vs. other grades. **C**:Forest plot of intubation time for tra-cheal intubation using the indirect laryngoscope compared with the Macintosh laryngoscope).

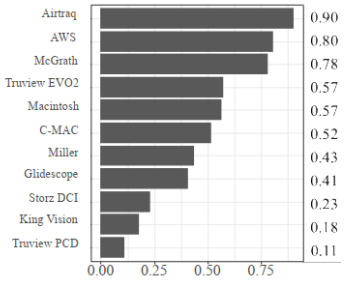


**(A)**

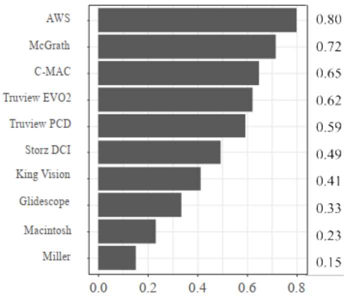


**(B)**

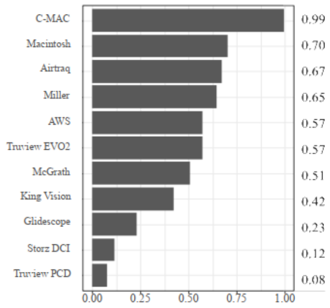


**(C)**

**Figure S14.** The results of subgroup analysis (**A** intubation failure, B glottic visualization, and **C** intubation time) by P score ranking.

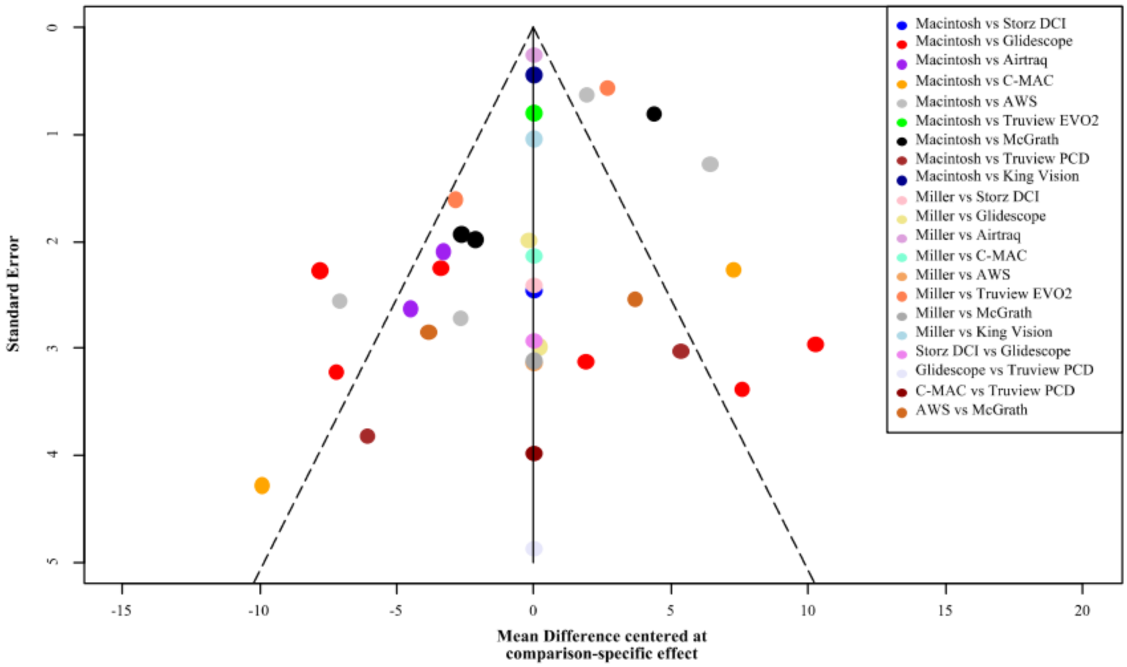


**(A)**

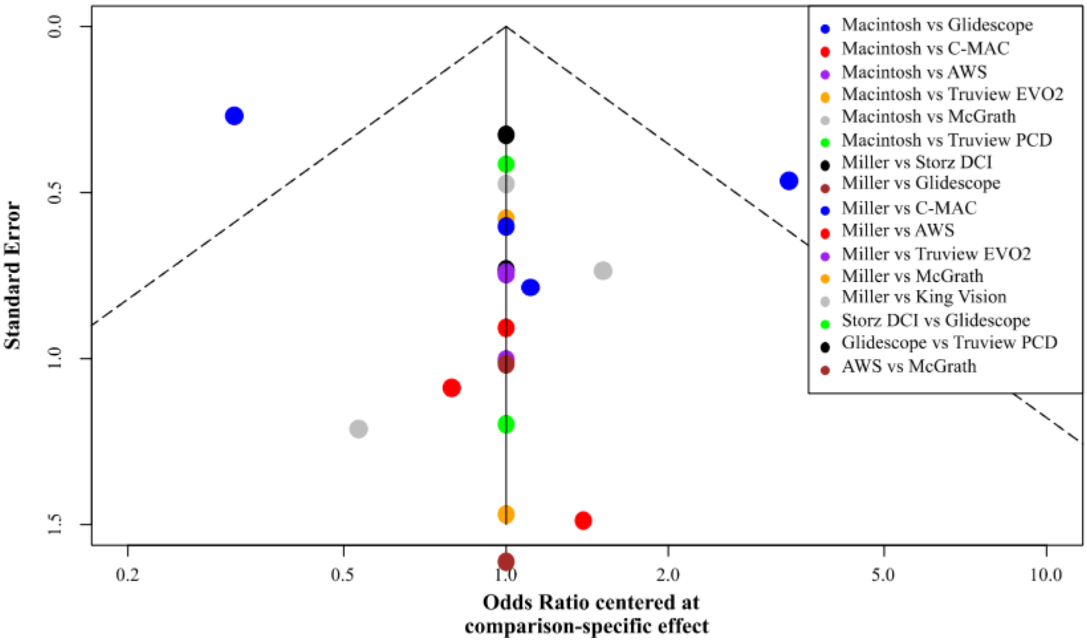


**(B)**

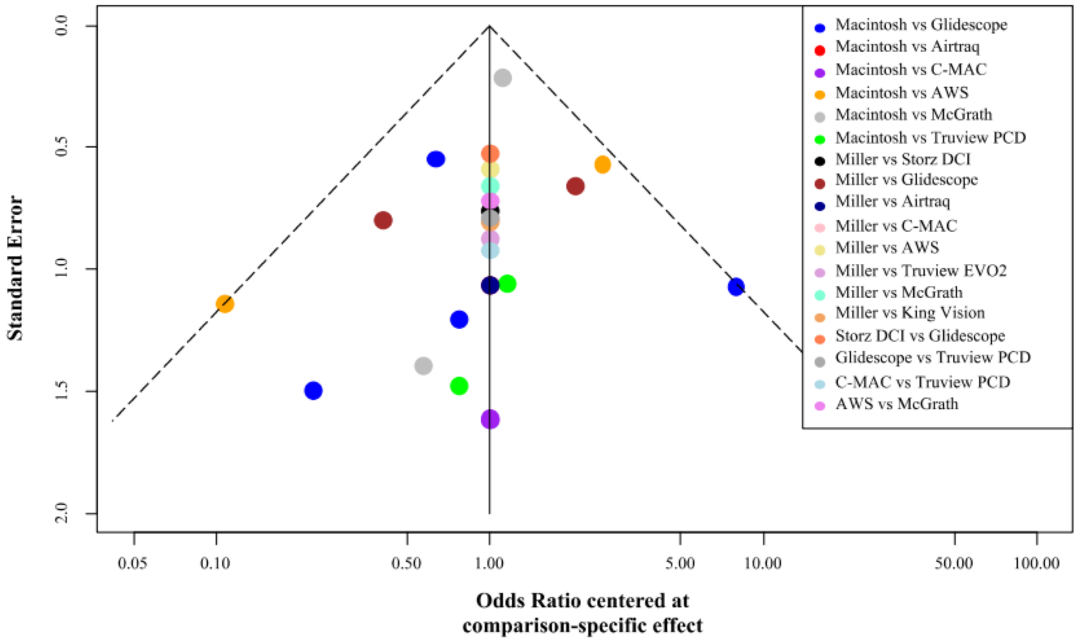


**(C)**

**Figure S15.** The results of funnel plot of the subgroup analysis (**A**：intubation failure, **B**:glottic visu-alization, and **C**:intubation time).
